# Comparative genomics and expression analysis of the *CIPK* gene family in rice (*Oryza sativa*) and foxtail millet (*Setaria italica*)

**DOI:** 10.3389/fpls.2025.1710663

**Published:** 2026-01-05

**Authors:** Zhao Hu, Run Qian, Fengpu Xie, Ziwei Wang, Pingmei Yan, Jing Yang

**Affiliations:** 1 College of Biological Sciences and Technology, Taiyuan Normal University, Taiyuan, China; 2 College of Life Science, Nanchang University, Nanchang, China; 3 School of Traditional Chinese Materia Medica, Shenyang Pharmaceutical University, Shenyang, China

**Keywords:** rice (*Oryza sativa*), foxtail millet (*Setaria italica*), CIPK, abiotic stress, comparative genomics

## Abstract

The *CIPK* (CBL-interacting protein kinase) gene family serves as a crucial component of calcium-mediated signaling pathways in plants, playing vital roles in abiotic stress responses and developmental regulation. Despite their functional importance, systematic comparative analyses of *CIPK* gene families between cereal species with distinct physiological adaptations remain limited. To address this knowledge gap, we present a comprehensive comparative genomic analysis of *CIPK* gene families in two ecologically and physiologically divergent cereal crops - rice (*Oryza sativa*, a C_3_ species adapted to aquatic environments) and foxtail millet (*Setaria italica*, a C_4_ species with superior drought tolerance). We identified 33 and 35 *CIPK* genes in rice and foxtail millet, respectively, revealing conserved synteny but distinct evolutionary trajectories. Chromosomal mapping showed uneven distributions of *CIPK* genes in both species. Segmental duplications may have significantly contributed to family expansion (20/33 genes in rice; 19/35 in foxtail millet). Phylogenetic analysis classified members into six clades in foxtail millet and rice. Structural analysis revealed clade-specific exon-intron patterns, with complex architectures (12–20 exons) in Clade I versus simplified structures (1–3 exons) in other clades. Comparative genomics identified 26 orthologous pairs, though some genes (e.g., *OsCIPK27*) lacked detectable orthologs, indicating species-specific gene loss. Notably, foxtail millet CIPK exhibited lower instability indices, which may indicate potential stability differences. In addition, foxtail millet showed complete conservation of calcium-sensing NAF domains, whereas rice showed one NAF-deficient member (OsCIPK4). Promoter analysis identified species-specific *cis*-element enrichment: rice *CIPK* were enriched in drought/ABA-responsive elements, whereas foxtail millet showed greater light-responsive motif diversity. Expression profiling revealed tissue-specific patterns, with foxtail millet displaying more leaf-preferential expression (10 genes vs. 7 in rice), potentially linked to C_4_ photosynthesis. Under abiotic stress conditions, rice *CIPK* genes exhibited strong responses to both salt and cold stressors, whereas foxtail millet *CIPK* showed greater responsiveness to drought, mirroring their ecological adaptations. These findings provide novel insight into the potential function and evolutionary diversification of calcium signaling components (*CIPK*) in C_3_ and C_4_ cereals. They also provide important potential targets for improving the stress resistance of cereal crops.

## 1 Introduction

Calcium (Ca^2+^) serves as a ubiquitous secondary messenger in plants, mediating responses to diverse environmental stimuli and developmental cues (Park and Shin, 2022). The decoding of Ca^2+^ signals is achieved through Ca^2+^-sensor proteins. Such as calcineurin B-like proteins (CBLs) and their interacting protein kinase (CIPK), which together form sophisticated signaling networks (Tang et al., 2020). The *CIPK* family plays a pivotal role in transducing Ca^2+^ signals by phosphorylating downstream targets, thereby regulating processes ranging from abiotic stress tolerance to nutrient homeostasis (Mao et al., 2023). Despite the extensive research conducted on *CIPK* in the model plant *Arabidopsis thaliana*, systematic comparisons of *CIPK* families in closely related cereal crops with distinct ecological adaptations remain limited, particularly between the water-intensive rice (*Oryza sativa*) and the drought-resistant foxtail millet (*Setaria italica*). Such comparative analyses are essential for understanding how calcium signaling networks have evolved to mediate species-specific stress adaptation strategies.

Rice and foxtail millet, both members of the Poaceae family, have evolved divergent adaptive strategies shaped by their distinct ecological niches. This divergence is most pronounced in their contrasting photosynthetic pathways. Rice uses the less efficient C_3_ pathway, resulting in lower water-use efficiency but higher photosynthetic rates under optimal conditions (Karki et al., 2013; Chen et al., 2020). In contrast, foxtail millet employs the more efficient C_4_ pathway, conferring greater water- and nitrogen-use efficiency, especially under high temperature and light intensity (Schuler et al., 2016; He et al., 2023). Despite the ecological and physiological importance of these two cereals, systematic comparative analyses of key gene families, such as those involved in calcium signaling, remain limited. In particular, the *CIPK* gene family, known to play a central role in abiotic stress response signaling, has not been comprehensively compared between these two species.

The fundamental differences in ecophysiological adaptation between rice and foxtail millet extend well beyond their distinct photosynthetic pathways. Rice, as a semi-aquatic species, has evolved specialized morphological and physiological adaptations to flooded conditions, most notably the development of aerenchyma tissue for efficient oxygen transport to submerged roots (Parlanti et al., 2011). However, this aquatic specialization has resulted in compromised drought tolerance, making rice particularly vulnerable to water deficit conditions. In stark contrast, foxtail millet has evolved as a xerophytic species with a suite of drought-adaptive traits, including: (i) an extensive and deep root system for enhanced water acquisition; (ii) rapid hormonal response systems for efficient stomatal regulation; and (iii) robust antioxidant mechanisms for reactive oxygen species detoxification (Liu et al., 2019; Amoah et al., 2023; Chang et al., 2024; Supriya et al., 2025). Rice and foxtail millet also differ markedly in salt tolerance - while rice is highly sensitive to NaCl concentrations above 50 mM (Shen et al., 2024), foxtail millet maintains growth at 150 mM NaCl (Saleem et al., 2023). Given the central role of *CIPK* in integrating calcium signaling with stress responses, comparative analysis of these gene families in rice and foxtail millet could reveal important insights into the evolution of stress adaptation mechanisms in C_3_ and C_4_ cereals.

In this study, we present a comprehensive comparative genomics analysis of *CIPK* gene families in rice and foxtail millet, combining phylogenetic, structural, and expression profiling approaches. We identified 33 *OsCIPK* and 35 *SiCIPK* genes and analyzed their: (i) chromosomal distribution and duplication histories; (ii) phylogenetic relationships and gene structure evolution; (iii) protein physicochemical properties and domain architectures; (iv) tissue-specific and stress-responsive expression patterns; and (v) promoter *cis*-element compositions. Our results reveal both deep conservation and striking divergence in *CIPK* family organization between these species, with foxtail millet showing expanded clades enriched in stress-responsive genes and rice exhibiting stronger salt and cold-induced expression shifts. These findings provide new insights into how calcium signaling networks have evolved to support environmental adaptation in cereals, offering potential targets for improving stress tolerance in crops through breeding or engineering strategies.

## 2 Materials and methods

### 2.1 Plant materials and growth conditions

The rice (ShijinB) and foxtail millet (JinGu21) plants were used in this study. Seeds were surface-sterilized with 5% (v/v) NaClO for 30 min at room temperature and thoroughly rinsed with double-distilled water. Subsequently, the seeds were germinated in distilled water at 30°C for two days. The germinated seeds were then transferred to 5-L containers filled with Kimura B nutrient solution and cultivated for an additional 10 days. The plants were maintained in a growth chamber under a 12-h light/12-h dark photoperiod (light intensity: approximately 200 µmol m^-2^ s^-1^) at 30°C during the light period and 28°C during the dark period. The nutrient solution was renewed every two days.

According to established protocols in plant stress physiology studies (Yoon et al., 2016; Kordrostami et al., 2017; Li et al., 2019; Ambadas et al., 2023; Lei et al., 2024), the stress treatments were administered as follows: 10-day-old seedlings were transferred to fresh Kimura B solution containing 100 mM NaCl for 2 h for salt stress; exposed to 4°C for 2 h for cold stress; and removed from nutrient solution for aerial desiccation for 2 h for drought stress.

### 2.2 RNA isolation and RT-qPCR analysis

RNA isolation and RT-qPCR analysis were performed according to established protocols (Hu et al., 2025). Briefly, total RNA was extracted using TRNzol Universal Reagent (TIANGEN, Cat. No. DP424). First-strand cDNA was synthesized from 1 µg of total RNA using the FastKing RT Kit (TIANGEN, Cat. No. KR116) according to the manufacturer’s instructions. Quantitative real-time PCR was conducted on a StepOnePlus™ Real-Time PCR System (Applied Biosystems) using Power SYBR™ Green Master Mix (Applied Biosystems). Each reaction was performed in a 20 µL volume following the recommended thermal cycling conditions. The Actin gene was used as an internal reference for normalization of target gene expression levels. Gene-specific primer pairs for RT-qPCR were designed using Primer-BLAST on the NCBI website. The sequences of the gene-specific primers utilized in this study are provided in **Supplementary Table S1**. The specificity of each primer pair was confirmed by observing a single peak in the melt-curve analysis. Primer amplification efficiency (E) was determined using a standard curve generated from a 5-fold serial dilution of a pooled cDNA sample. The slope of the standard curve was used to calculate efficiency according to the formula: E = (10^(-1/slope) - 1) × 100%. All primers used in this study had an efficiency between 100% and 110% (**Supplementary Table S1**), with correlation coefficients (R²) greater than 0.990. All experiments included three independent biological replicates per sample.

### 2.3 Genome-wide identification of *CIPK* gene family members

The identification of *CIPK* gene family members was conducted through an integrated computational approach combining homology-based searches and domain architecture analysis. Initial candidate genes were identified using BLASTp analysis (E-value ≤ 1e-5) against the rice and foxtail millet proteome databases, employing experimentally characterized CIPK protein sequences from *Arabidopsis thaliana* and *Oryza sativa* as queries. Simultaneously, profile hidden Markov model (HMM) searches were performed using HMMER v3.3.2, with the canonical models for the serine/threonine protein kinase domain and NAF domains obtained from the Pfam database (http://pfam.xfam.org/). The resulting candidate sequences were subjected to rigorous domain architecture validation using InterProScan (https://www.ebi.ac.uk/interpro/search/sequence/) and SMART databases (https://smart.embl.de/smart/change_mode.cgi).

### 2.4 Chromosome localization and gene structure analysis

The *CIPK* gene sequences in rice and foxtail millet were retrieved from the NCBI database (https://www.ncbi.nlm.nih.gov/) and the structures were checked using IGV-GSAman (v0.9.51). Chromosomal localization and gene structure analysis were performed using TBtools. The physical positions of the *CIPK* genes were determined for chromosomal mapping based on the Rice Genome Annotation Project data (https://rice.uga.edu/) or the Setaria Comprehensive Database (http://111.203.21.71:8000/index.html), while the gene structures were analyzed by comparing the coding sequences with the corresponding genomic DNA sequences.

### 2.5 Phylogenetic relationships and gene duplication analysis

The amino acid sequences of OsCIPK and SiCIPK proteins were aligned to generate a phylogenetic tree using MEGA12 software. The neighbour-joining (with the p-distance model) and maximum-likelihood (with the Jones-Taylor-Thornton model) methods were employed, with 1000 replications for bootstrapping. Gene duplication analysis using MCScanX identified both whole-genome and tandem duplication events. The Ka/Ks ratios for the duplicated gene pairs were calculated using the Nei-Gojobori method (Jukes-Cantor model) implemented in KaKs_Calculator 3.0.

### 2.6 Conserved domain and conserved motif analysis

Conserved domain analysis was performed using the Pfam (http://pfam.xfam.org/) and InterProScan (https://www.ebi.ac.uk/interpro/search/sequence/) websites. MEME-based motif discovery was performed with the following parameters: ten motifs with a width of 6–50 and an E-value of e-10 were identified via the MEME website (http://meme-suite.org/).

### 2.7 Subcellular localization and *cis*-elements analysis

WoLF PSORT (https://wolfpsort.hgc.jp/) online website was used to analyze subcellular localization. The 2.0 kb promoter sequences located in the upstream region of each *OsCIPK* or *SiCIPK* gene were retrieved from the Rice Genome database and Setaria Genome database using the TBtools software. A systematic analysis of these promoter regions was conducted using the PlantCARE database (http://bioinformatics.psb.ugent.be/webtools/plantcare/html/) to identify putative *cis*-regulatory elements.

### 2.8 RNA-seq data analysis

The RNA-seq data presented in this study can be found in NCBI website (https://www.ncbi.nlm.nih.gov/). The names of the repository/repositories and accession number(s) can be found at: BioProject ID (**Supplementary Table S2**): PRJNA482217, PRJNA827493, PRJNA1037192, PRJNA306542. PRJNA1155684, PRJNA805389 and PRJNA767196. The RNA-seq data were processed according to (Hu et al., 2024). In brief, the raw reads were quality-trimmed using Trimmomatic and aligned to the NIP reference genome (for rice) and YuGu1 reference genome (for foxtail millet) using STAR software with default parameters. Gene expression levels were quantified as FPKM using StringTie (v2.2.1). For differential expression analysis under stress conditions, the DESeq2 package (v1.38.3) in R was employed. Genes with an absolute log_2_ fold change |log_2_FC| > 1 and a false discovery rate (FDR) adjusted p-value (padj) < 0.05 were considered differentially expressed. All analyses were based on three independent biological replicates. Additionally, it should be noted that, while within-study normalization was performed using the standard DESeq2 workflow, no cross-dataset batch correction was applied to the integrated RNA-seq data from different BioProjects due to substantial variations in experimental designs and cultivars.

### 2.9 Statistical analysis

All experimental data are presented as mean ± standard deviation (SD) from three independent biological replicates. Statistical significance was determined using Student’s *t*-test, with a significance threshold of *p* < 0.05. All analyses were performed using GraphPad Prism software (version 9).

## 3 Results

### 3.1 Chromosomal localization and duplication analysis of *CIPK* genes in rice and foxtail millet

**Figure 1:**
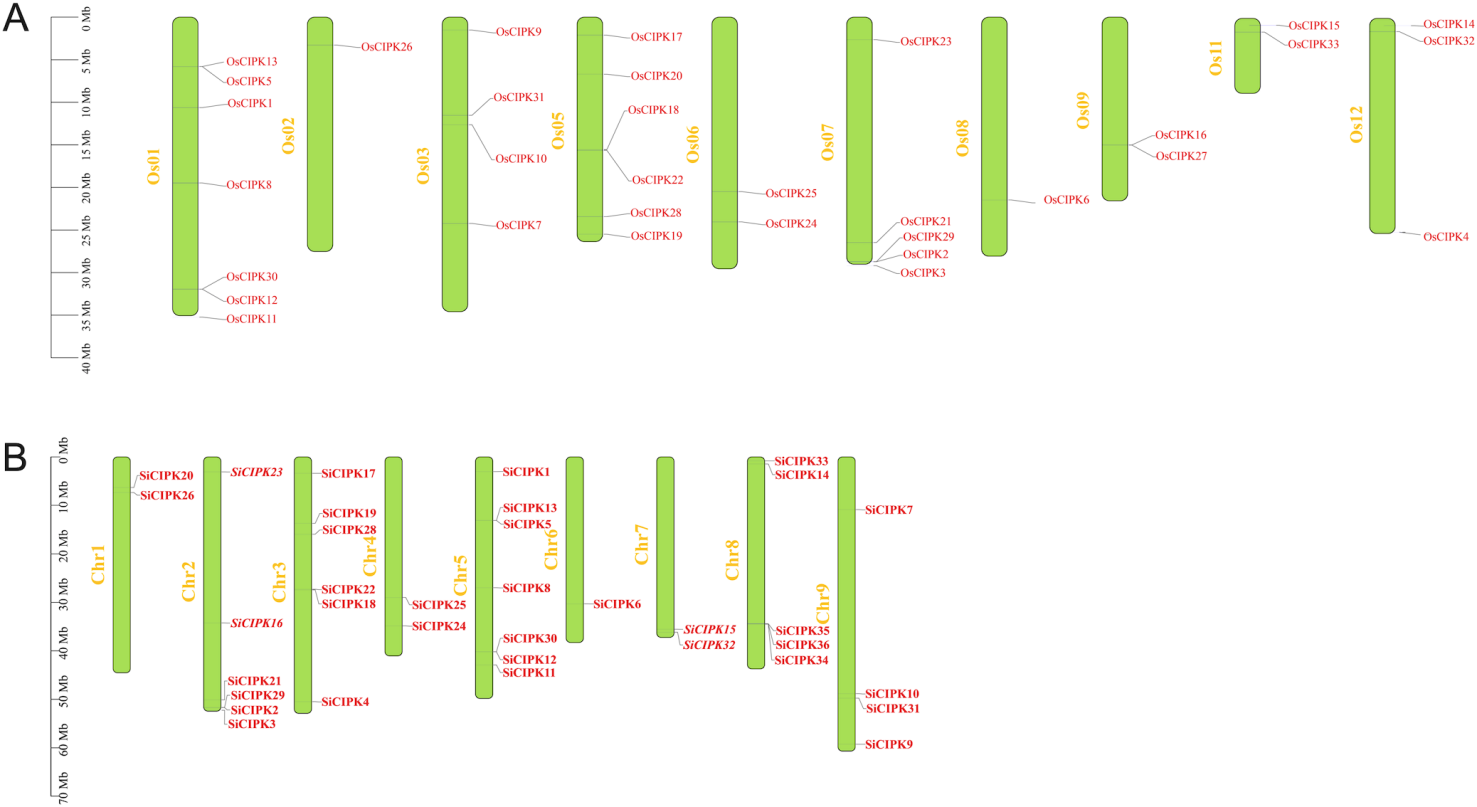
Distribution of *CIPK* genes in rice **(A)** and foxtail millet **(B)** chromosomes. Only show the chromosomes where located the *CIPK* gene. The yellow font represents chromosome numbers and green arc length indicate chromosome size.

Comprehensive mapping of the *CIPK* gene families in both species revealed uneven distributions across their respective genomes. In rice, the 33 *OsCIPK* genes were localized to 10 out of 12 chromosomes. Chromosomes 1 and 5 harboring the highest numbers (7 and 6 genes, respectively), while chromosomes 2 and 8 contained only one member each (**Figure 1A**; **Supplementary Table S3**). Notably, gene clusters were observed in specific chromosomal regions, such as the proximal region of chromosome 7, which housed *OsCIPK2*, *OsCIPK3* and *OsCIPK29*. To comprehensively identify *CIPK* family members in foxtail millet, we performed a genome-wide search using a combined strategy of BLASTp and HMM profiling. This approach led to the identification of 35 non-redundant *SiCIPK* genes (**Figure 1B**; **Supplementary Table S3**). To facilitate comparative and functional studies, each of the 35 identified *SiCIPK* genes was systematically named according to the established nomenclature of their orthologs in rice (**Supplementary Figure S1**). In foxtail millet, the 35 *SiCIPK* genes were distributed unevenly across 9 chromosomes, with chromosome 5 containing the highest number (7 genes) and chromosome 6 possessing only one member (**Figure 1B**). A prominent cluster was identified on chromosome 8, including *SiCIPK34*, *SiCIPK35* and *SiCIPK36*. The non-random distribution of *CIPK* genes in both species suggests the occurrence of gene duplication events during evolution. To confirm this, we analyzed duplication events using MCScanX. In rice, 11 segmental duplication events were identified, involving 20 out of 33 *OsCIPK* genes (**Supplementary Figure S2A**), indicating that large-scale genomic duplications may have significantly contributed to the family’s expansion. Similarly, foxtail millet exhibited 14 segmental duplication events, encompassing 19 of the 35 *SiCIPK* genes (**Supplementary Figure S2B**). Furthermore, Ka/Ks analysis revealed that the vast majority of duplicated *CIPK* genes exhibited Ka/Ks ratios significantly below 1 (**Supplementary Table S4**), indicating they underwent intense purifying selection consistent with their critical roles in calcium signaling.

### 3.2 Phylogenetic relationships and structure analysis of *CIPK* gene families in rice and foxtail millet

**Figure 2:**
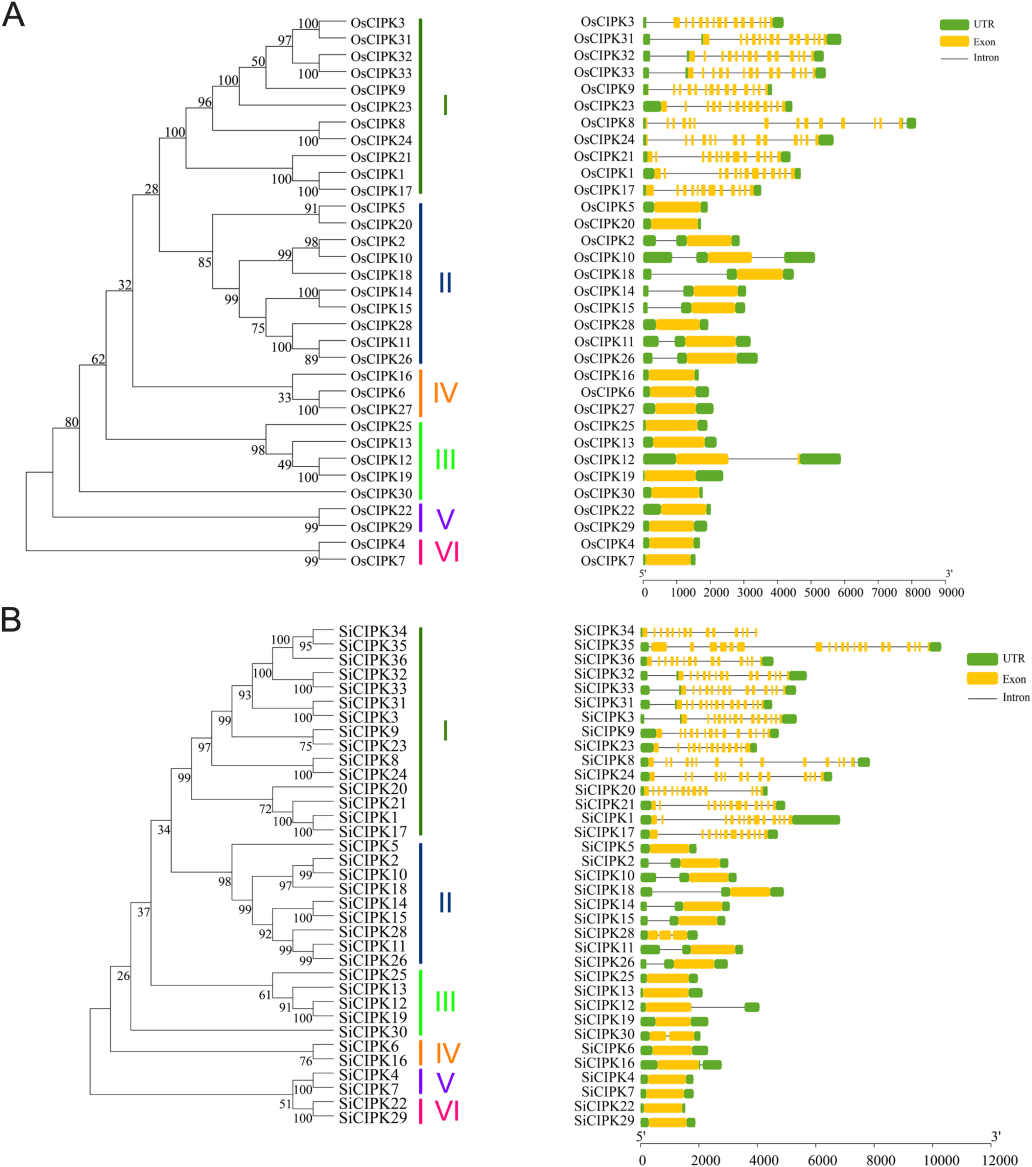
Phylogenetic relationships and gene structure analysis of *CIPK* in rice **(A)** and foxtail millet **(B)**. Phylogenetic relationships analysis by neighbour-joining (NJ) method. Phylogenetic relationships and gene structure analysis utilize protein sequences and DNA sequences, respectively. Green boxes, yellow boxes and black lines represent UTR, Exon and Introns respectively.

The phylogenetic relationships of *CIPK* genes were investigated through full-length protein sequences from both rice and foxtail millet by neighbour-joining and maximum-likelihood methods (**Figure 2**; **Supplementary Figure S3**). The resulting phylogenetic trees revealed that the *CIPK* families in both species segregated into six distinct clades (Clades I-VI) (**Figure 2**), demonstrating deep evolutionary conservation of this gene family. In rice, the 33 *OsCIPK* genes showed uneven distribution across clades, with Clade I (11 genes) and Clade II (10 genes) representing the most expanded groups, while Clades V and VI contained only two members each (**Figure 2A**). A similar pattern was observed in foxtail millet, where the 35 *SiCIPK* genes also clustered into six corresponding clades. Among them, Clade I contacted 15 genes, whereas the Clades IV-VI contacted only 2–3 genes each (**Figure 2B**). These patterns suggest potential lineage-specific gene loss or functional specialization in the smaller clades.

Gene structure analysis revealed both conserved and divergent architectures across the *CIPK* families. In both species, Clade I members typically contained 12–20 exons, maintaining a complex structure that likely represents the ancestral form. In contrast, genes in other clades exhibited simpler structures, with rice Clades II-VI containing 1–2 exons and foxtail millet Clades II-VI showing slightly more variation (1–3 exons) (**Figure 2**). This clade-specific conservation of exon-intron patterns strongly supports the phylogenetic classification and suggests structural evolution may be linked to functional diversification. Comparative analysis between the two species identified 26 orthologous *CIPK* gene pairs (**Supplementary Figure S1**), indicating substantial syntenic conservation from their shared ancestor. However, some genes like *OsCIPK27* lacked detectable orthologs in foxtail millet, suggesting species-specific gene loss or rapid divergence after speciation. Notably, in contrast to Clade I, the gene structural simplification observed in other clades, particularly the reduction to 1–3 exons, implies possible neofunctionalization events.

### 3.3 Diverse physicochemical properties of the CIPK protein family in rice and foxtail millet

**Figure 3:**
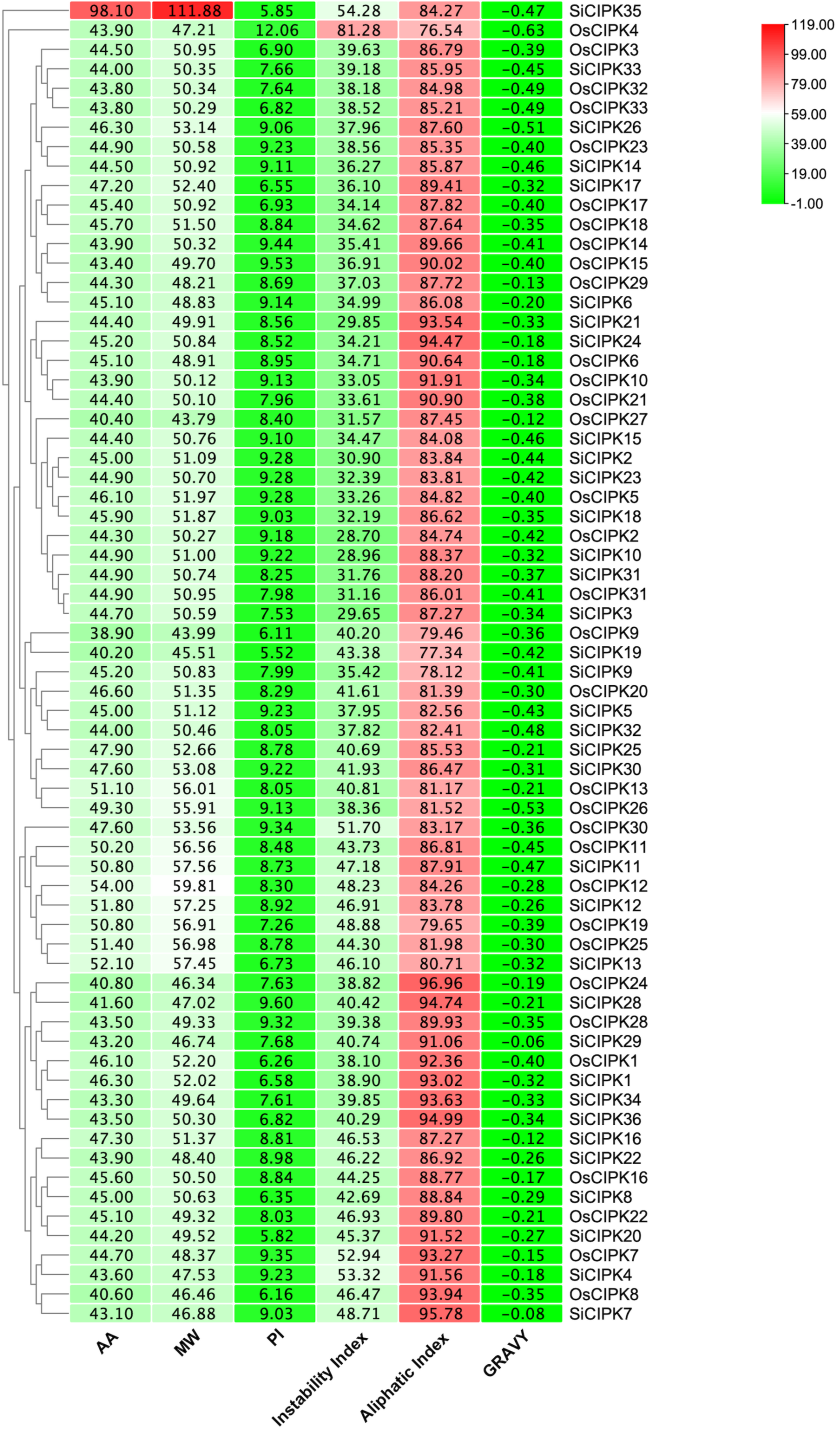
Protein information of OsCIPK and SiCIPK family. AA, amino acid number (The values shown in the “AA” bar graph represent one-tenth of the actual amino acid count to facilitate visual comparison with other metrics); MW, molecular weight (kDa); pI, isoelectric point; GRAVY, the grand average of hydropathy.

Comprehensive analysis of the physicochemical properties of CIPK proteins in rice and foxtail millet revealed substantial diversity in molecular characteristics (**Figure 3**; **Supplementary Table S5**). The 33 OsCIPK proteins in rice exhibited molecular weights ranging from 43.79 to 59.81 kDa, with OsCIPK12 (59.81 kDa) and OsCIPK27 (43.79 kDa) representing the largest and smallest members, respectively. Similarly, the 35 SiCIPK proteins in foxtail millet showed a broader molecular weight range (45.51-111.9 kDa). Among them, SiCIPK35 (111.88 kDa) was exceptionally large compared to other family members. The isoelectric points (pI) of OsCIPK varied from 6.11 to 12.06, indicating both acidic and basic proteins. SiCIPK displayed a narrower pI range (5.52-9.60), suggesting more consistent charge characteristics in foxtail millet. The instability index analysis revealed that most CIPK proteins in both species were relatively stable, with values below 50. However, OsCIPK4 (81.28) and SiCIPK4 (53.32) showed notably higher instability indices, suggesting these proteins may have distinct regulatory mechanisms. Aliphatic index values, which reflect thermal stability, ranged from 76.54 to 96.96 in rice and 77.34 to 95.78 in foxtail millet, indicating generally good thermostability across the protein family. The grand average of hydropathy (GRAVY) scores were consistently negative for all CIPK members in both species (-0.63 to -0.12 in rice; -0.51 to -0.06 in foxtail millet), confirming their hydrophilic nature and likely soluble cytoplasmic localization. In addition, the exceptional size of SiCIPK35 (111.88 kDa) suggests possible gene fusion or unique domain architecture in this particular member. These diverse physicochemical characteristics likely reflect functional specialization within the CIPK family. Variations in molecular weight, stability, and hydrophobicity potentially influencing subcellular localization and stress response mechanisms in each species.

### 3.4 Integrated analysis of OsCIPK and SiCIPK protein structures and subcellular localization

**Figure 4:**
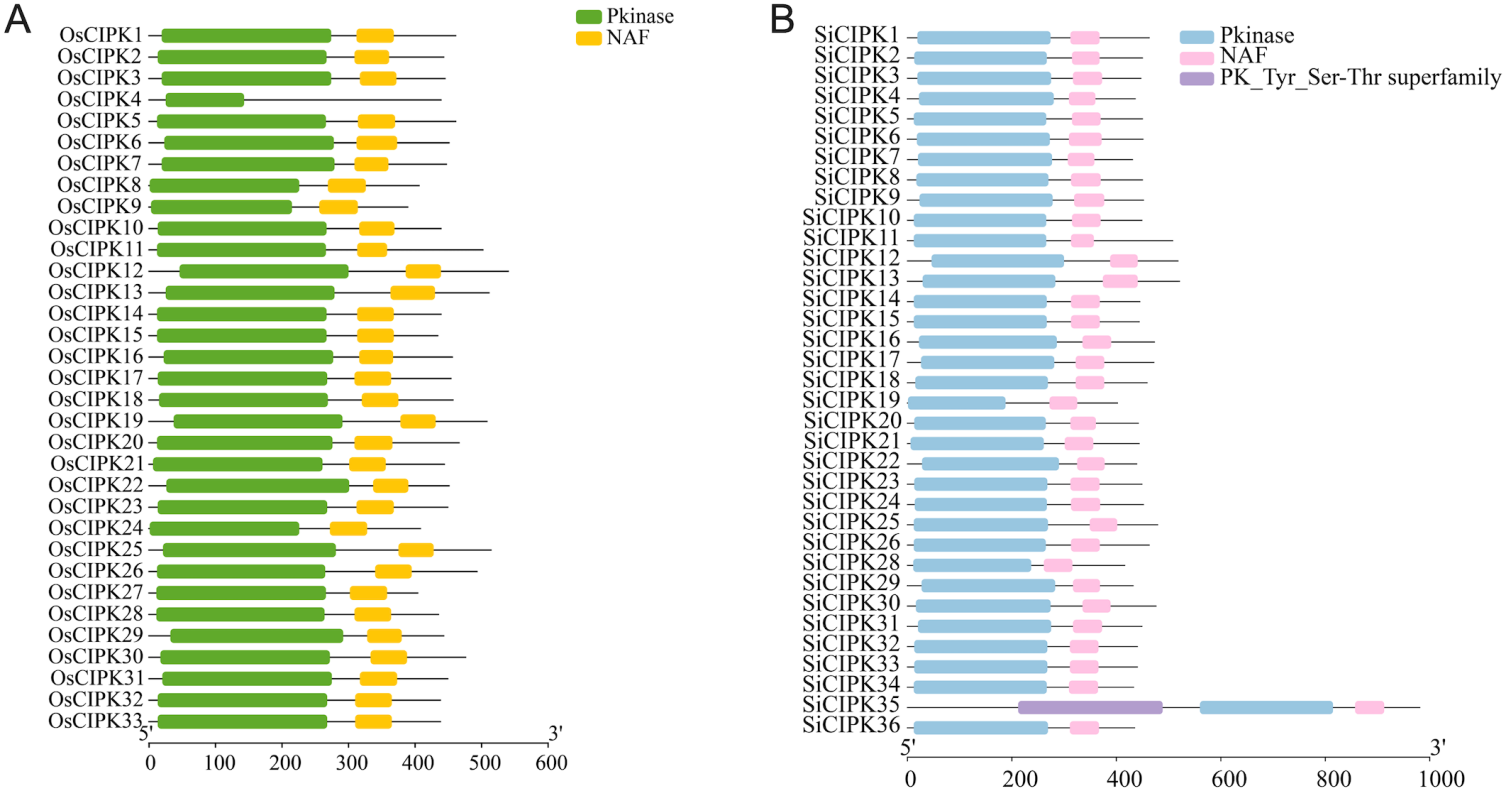
Conserved domain analysis of the OsCIPK **(A)** and SiCIPK **(B)**.

**Figure 5:**
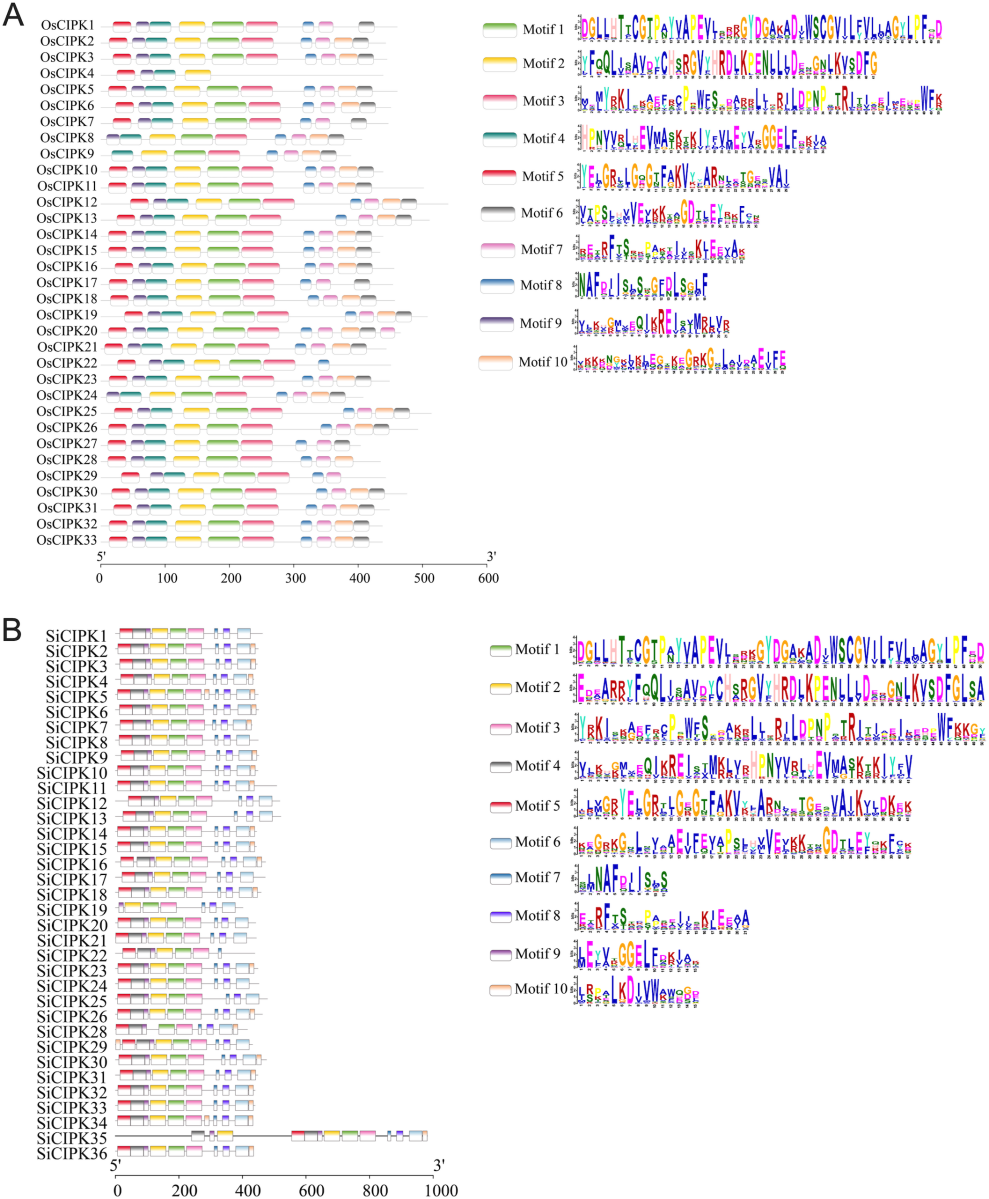
Conserved motif analysis of the OsCIPK **(A)** and SiCIPK **(B)**. Different Motifs are represented by different colors.

To elucidate the structural basis of CIPK function, we systematically analyzed the conserved structural features in both OsCIPK and SiCIPK protein families by Pfam database (El-Gebali et al., 2019). All 33 OsCIPK and 35 SiCIPK members contained the canonical serine/threonine protein kinase domain (PKinase) with highly conserved catalytic motifs (HRDxxxxN and DFG) (Hrabak et al., 2003; Kornev et al., 2006), confirming their conserved phosphotransferase activity across both species (**Figures 4**, **5**). Notably, 32 OsCIPK and all 35 SiCIPK possessed the NAF domain essential for interaction with CBL calcium sensors (**Figure 4B**). This conservation pattern suggests strong evolutionary pressure to maintain calcium signaling capability in both species. However, the complete retention of NAF domains in all SiCIPK compared to the single exception in rice (OsCIPK4) points to potential species-specific functional diversification (**Figure 4A**).

**Figure 6:**
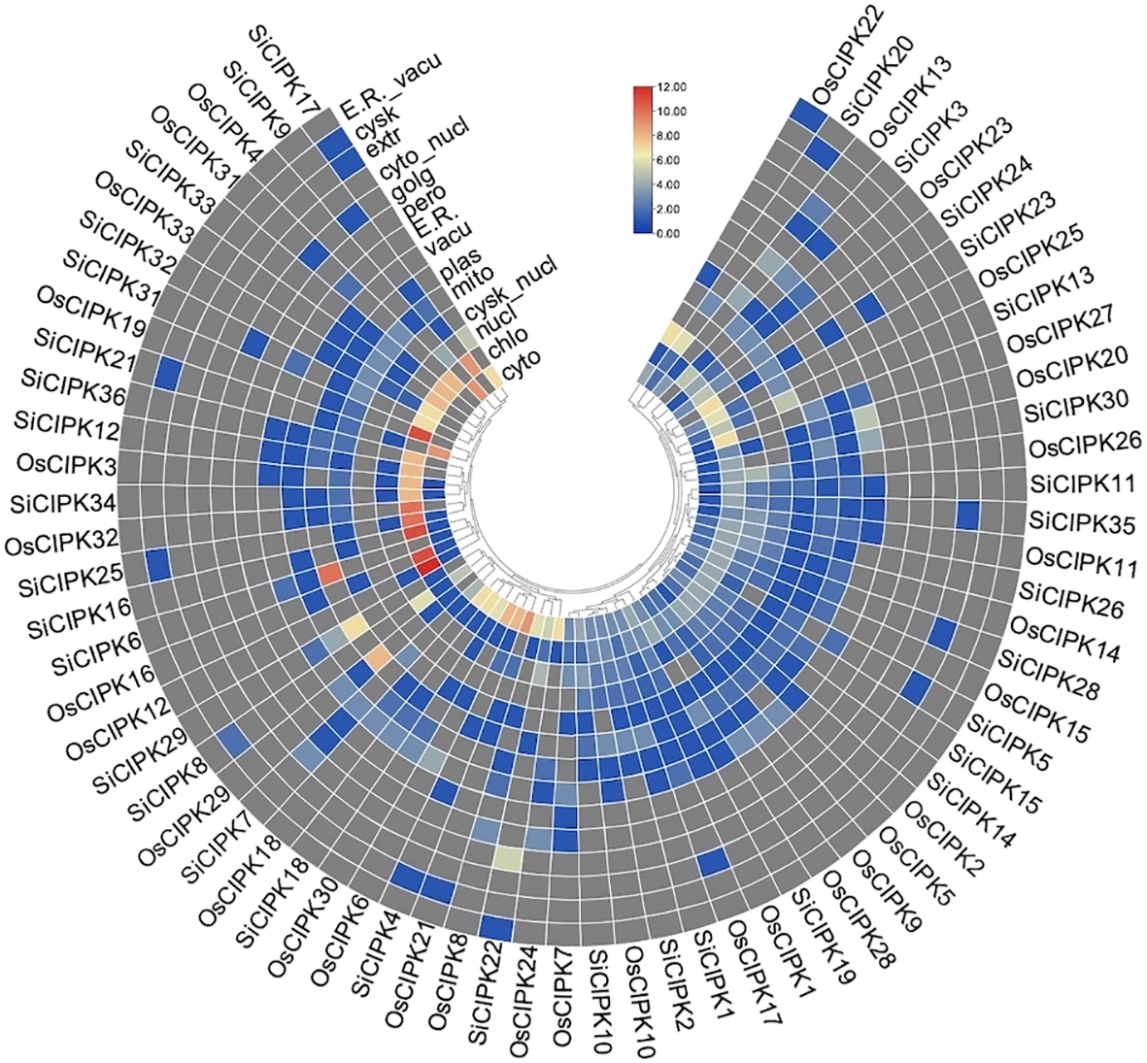
The subcellular localization prediction analysis of OsCIPK and SiCIPK proteins. The color scale represents subcellular localization prediction scores from low (blue) to high (red).

Subcellular localization predictions revealed distinct compartmentalization preferences for CIPK proteins in both species (**Figure 6**). Chloroplast localization was particularly prevalent, with multiple members in each species showing strong chloroplast signals (OsCIPK3, OsCIPK12, OsCIPK16, OsCIPK19, OsCIPK31, OsCIPK21 and OsCIPK32; SiCIPK9, SiCIPK12, SiCIPK16, SiCIPK25, SiCIPK33, SiCIPK34 and SiCIPK36), indicating their potential involvement in photosynthesis-related calcium signaling. Cytosolic-enriched proteins (OsCIPK4, OsCIPK6 and OsCIPK21; SiCIPK4 and SiCIPK21) were also identified, suggesting roles in cytoplasmic calcium signaling cascades. Interestingly, several members in both species exhibited dual or multiple localization patterns (OsCIPK4, OsCIPK22 and OsCIPK26; SiCIPK8, SiCIPK13, SiCIPK17, SiCIPK22, SiCIPK29 and SiCIPK30), which may enable participation in cross-compartmental signaling networks. Particularly noteworthy was the complete conservation of subcellular localization between OsCIPK18 and its foxtail millet ortholog SiCIPK18, suggesting strong evolutionary constraint on the cellular targeting of this protein pair.

These structural and localization analyses collectively demonstrate that while the core kinase function is strictly conserved, both species have evolved distinct subcellular targeting patterns that likely reflect adaptations to their specific physiological requirements. The prevalence of chloroplast-localized members in particular suggests an important conserved role for CIPK in coordinating calcium signaling with photosynthetic processes in these cereal crops.

### 3.5 Expression pattern analysis of *CIPK* in rice and foxtail millet

**Figure 7:**
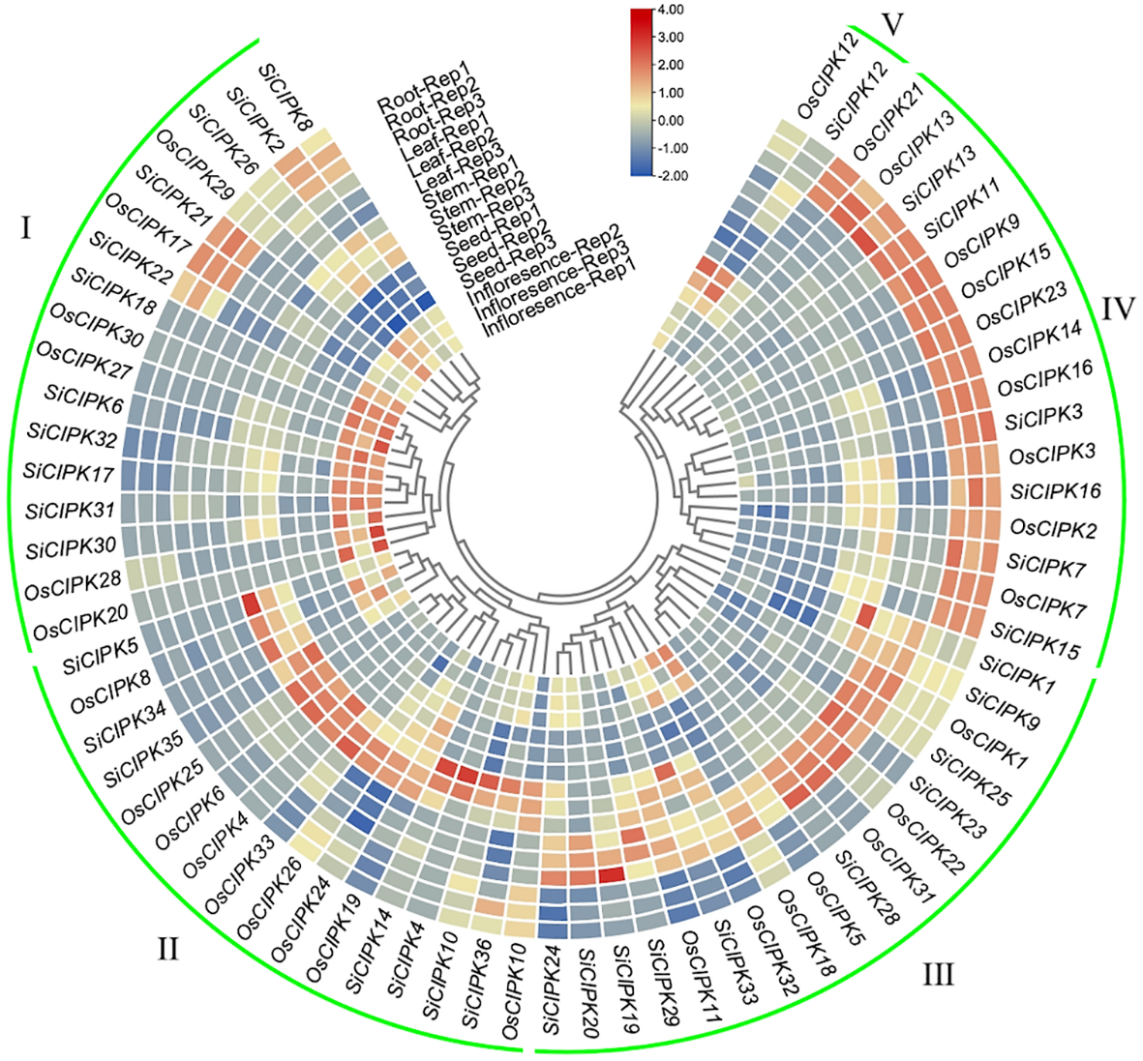
Heatmap of *CIPK* gene expression across rice and foxtail millet tissues. The expression data were obtained from the Rice Genome Annotation Project (https://rice.uga.edu/index.shtml) and the Setaria comprehensive database (http://111.203.21.71:8000/index.html). The heatmap was generated based on row-wise Z-score normalized log_2_(FPKM + 1) values. The color scale represents relative expression levels from low (blue) to high (red).

To investigate the functional specialization of *OsCIPK* and *SiCIPK* genes, we analyzed their tissue-specific expression patterns through hierarchical clustering (**Figure 7**). The analysis identified five distinct clusters with clear tissue specificity. Cluster I, which was primarily associated with inflorescence tissues, was markedly enriched in *SiCIPK* genes, accounting for 64.7% of the cluster. In contrast, cluster IV, which exhibited predominant expression in root tissues, was dominated by *OsCIPK* genes, comprising 62.5% of the cluster. This striking divergence in tissue-specific gene enrichment may reflect distinct evolutionary adaptations or functional specializations in reproductive and root development between the two species. In addition, cluster III was primarily linked to leaf-enriched expression. A comparative analysis showed that foxtail millet exhibited a broader set of these leaf-expressed *CIPK* than rice (10 in foxtail millet vs 7 in rice), suggesting potential differences in their photosynthetic strategies. A stem tissue-associated expression pattern defined Cluster II, which included nine stem-predominant genes (e.g., *OsCIPK4*, *OsCIPK6*) in rice and seven (e.g., *SiCIPK4*, *SiCIPK5*) in foxtail millet. Interestingly, certain orthologous pairs, including *OsCIPK2*/*SiCIPK2*, *OsCIPK13*/*SiCIPK13* and *OsCIPK3*/*SiCIPK3*, displayed highly similar expression profiles and co-clustered together. This suggests that the tissue-specific expression patterns of these genes are evolutionarily conserved between rice and foxtail millet. Conversely, divergent expression in some orthologs (e.g., root-predominant *OsCIPK23* vs leaf-preferential *SiCIPK23*) indicates possible subfunctionalization after species divergence.

### 3.6 Promoter analysis of *CIPK* in rice and foxtail millet

**Figure 8:**
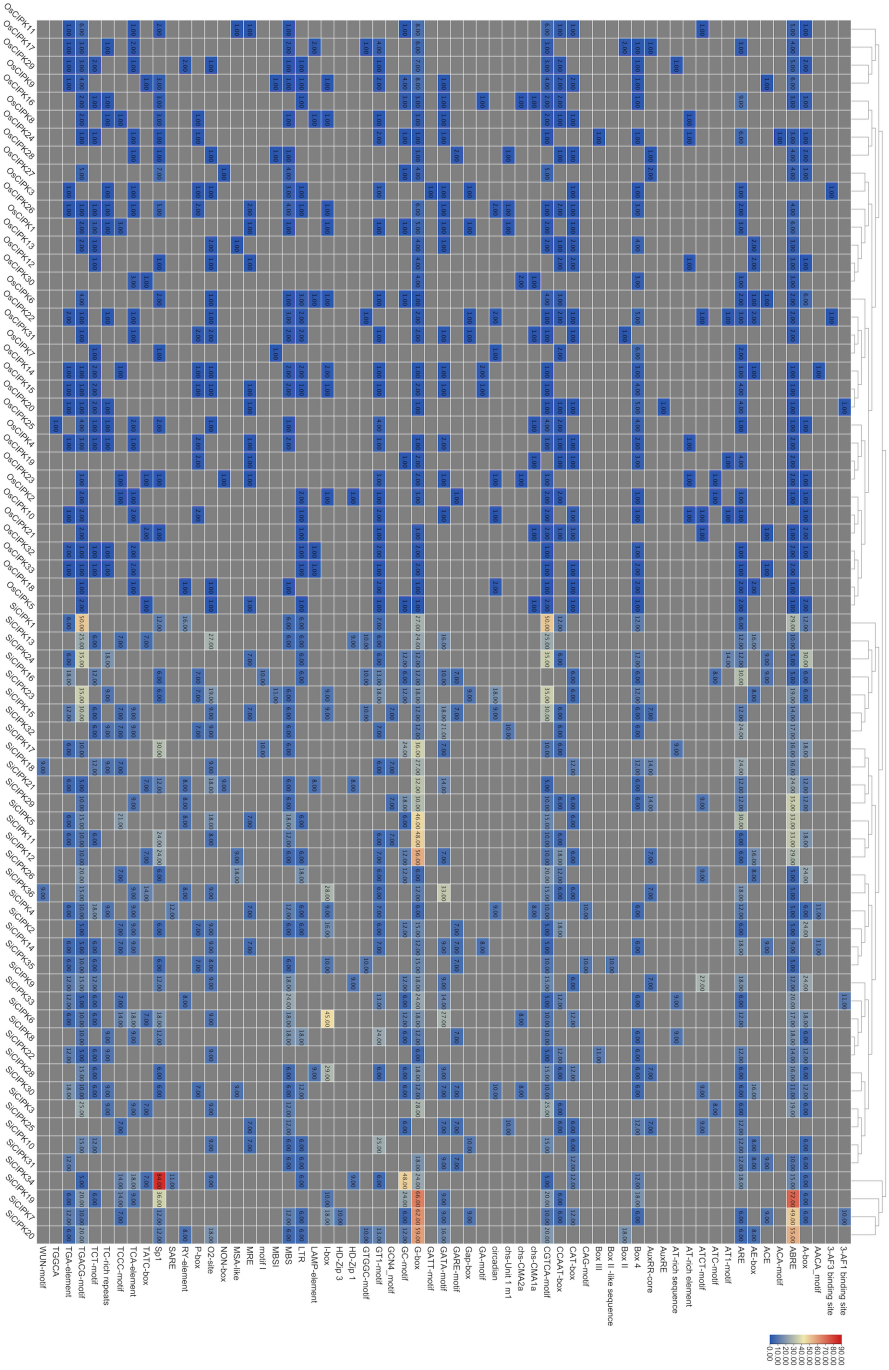
The number of *cis*-elements in each *OsCIPK* or *SiCIPK* gene promoter. Promoter sequences (-2Kb) of *CIPK* were analyzed by PlantCARE. The color scale represents the number of *cis*-elements from small (blue) to great (red).

As central regulators of gene expression, promoters play a critical role in determining transcriptional patterns. To investigate the potential regulatory basis for the differential expression of *SiCIPK* and *OsCIPK* genes across tissues, we systematically analyzed their promoter *cis*-element compositions. This analysis revealed distinct *cis*-regulatory architectures associated with stress adaptation, hormonal signaling, and developmental processes (**Figure 8**; **Supplementary Figure S4**). Examination of the 2000 bp promoter regions of all 33 *OsCIPK* genes in rice identified 893 *cis*-elements representing 56 functional categories (**Figure 8**). ABA-responsive ABRE elements were detected in 29 *OsCIPK* promoters. Among these, *OsCIPK1* and *OsCIPK9* exhibited the highest density (six copies each), suggesting a potential role in abscisic acid-mediated stress responses. Drought-inducible MBS motifs were present in 54% of *OsCIPK* genes, particularly enriched in *OsCIPK26* and *OsCIPK27* (four copies each). Cold-responsive LTR elements were most abundant in *OsCIPK6*. Furthermore, 81.8% of *OsCIPK* promoters contained MeJA-responsive CGTCA/TGACG motifs. The highest copy numbers (five to six) were observed in *OsCIPK11* and *OsCIPK27* harboring, indicating potential involvement in jasmonate signaling pathways. The analysis of 35 *SiCIPK* promoters in foxtail millet revealed a wide variety of *cis*-elements associated with environmental and developmental regulation (**Figure 8**). Light-responsive motifs, such as the G-box (CACGTG and CACGTT), Sp1 (GGGCGG) and GT1 motif (GGTTA and GGTTAAT), were widespread, suggesting a potential involvement in photomorphogenesis. Hormone-related elements were also prevalent: MeJA-responsive CGTCA/TGACG motifs were frequent, with multiple copies identified in *SiCIPK1*, while ABA-responsive ABRE elements were abundant in *SiCIPK11* and *SiCIPK20*. Auxin-signaling TGA elements (AACGAC) were identified in *SiCIPK1*, *SiCIPK14* and *SiCIPK22*, suggesting they may be involved in auxin-mediated growth regulation. Motifs involved in stress adaptation included LTR elements (CCGAAA) in *SiCIPK1*, *SiCIPK10* and *SiCIPK13* (cold response); ARE motifs (AAACCA) in *SiCIPK12* and *SiCIPK16* (hypoxia response); and MBS sites (CAACTG) in *SiCIPK1*, *SiCIPK20* and *SiCIPK33* (drought tolerance). Seed-specific RY elements (CATGCATG) in *SiCIPK20* and *SiCIPK21* hinted at potential roles in seed development.

Comparative analysis revealed species-specific differences in promoter architectures. While both *OsCIPK* and *SiCIPK* promoters were enriched in stress- and hormone-responsive elements, foxtail millet *SiCIPK* promoters exhibited greater diversity in light-responsive motifs, possibly reflecting adaptations to distinct environmental conditions. In contrast, rice *OsCIPK* promoters showed stronger enrichment in drought- and ABA-related elements, consistent with their known roles in stress responses under paddy field cultivation. To further explore the *cis*-regulatory divergence between rice and foxtail millet *CIPK* genes, we performed cluster analysis (**Figure 8**). Interestingly, the promoter elements of *OsCIPK* and *SiCIPK* genes grouped into two distinct clusters. All *OsCIPK* genes clustered together into one group, while all *SiCIPK* genes formed another separate cluster. Despite the conserved functions of many *CIPK* genes, this pronounced species-level divergence in promoter architecture suggests that the regulatory mechanisms governing their expression may have diverged significantly after the evolutionary split between rice and foxtail millet.

### 3.7 Comparative analysis of *OsCIPK* and *SiCIPK* expression patterns under abiotic stress conditions

**Figure 9:**
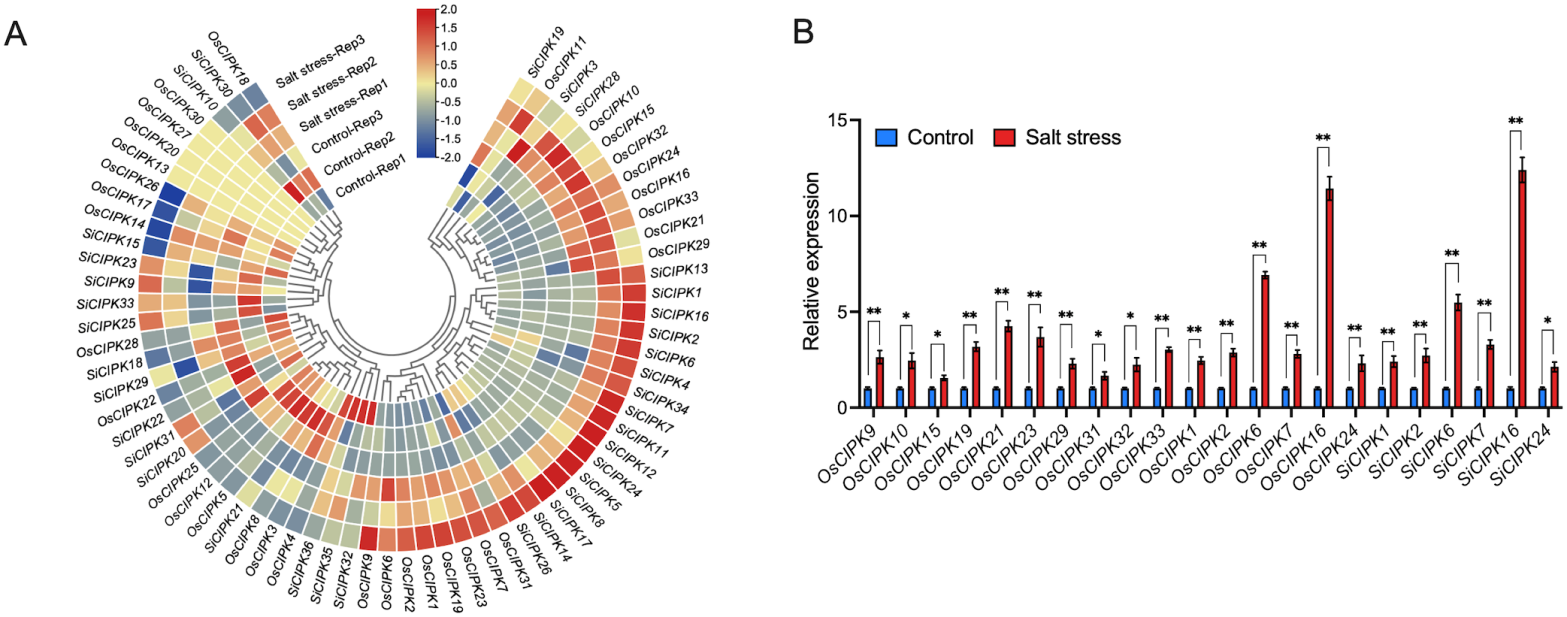
Expression analysis of the *CIPK* genes under salt stress conditions. **(A)** The expression analysis of *CIPK* genes in response to salt stress by RNA-seq. The heatmap was generated based on row-wise Z-score normalized log_2_(FPKM + 1) values. The color scale represents relative expression levels from low (blue) to high (red). **(B)** The expression analysis of *CIPK* genes in response to salt stress by qRT-PCR. n=3 biologically independent samples. The error bars represent ± SD. *p < 0.05, and **p < 0.01 compared to the Control (*t*-test).

**Figure 10:**
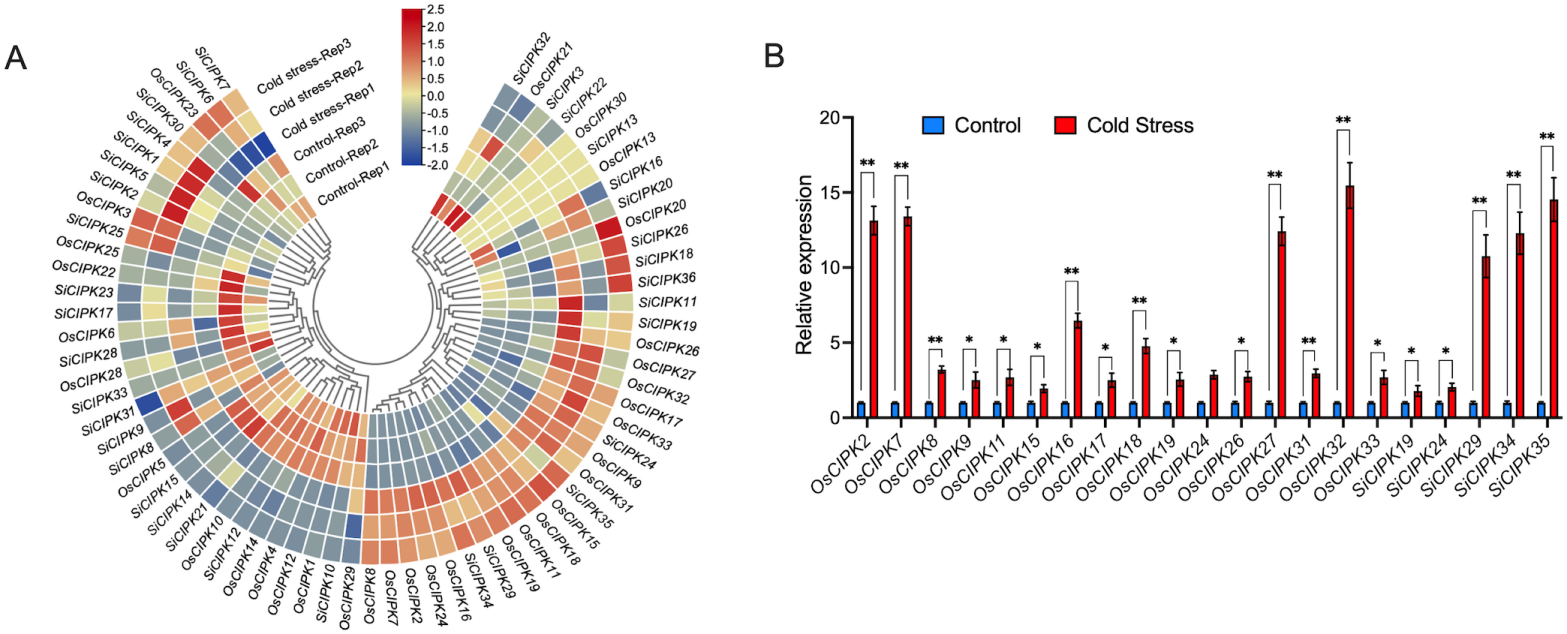
Expression analysis of the *CIPK* genes under cold stress conditions. **(A)** The expression analysis of *CIPK* genes in response to cold stress by RNA-seq. The heatmap was generated based on row-wise Z-score normalized log_2_(FPKM + 1) values. The color scale represents relative expression levels from low (blue) to high (red). **(B)** The expression analysis of *CIPK* genes in response to cold stress by qRT-PCR. n=3 biologically independent samples. The error bars represent ± SD. *p < 0.05, and **p < 0.01 compared to the Control (*t*-test).

**Figure 11:**
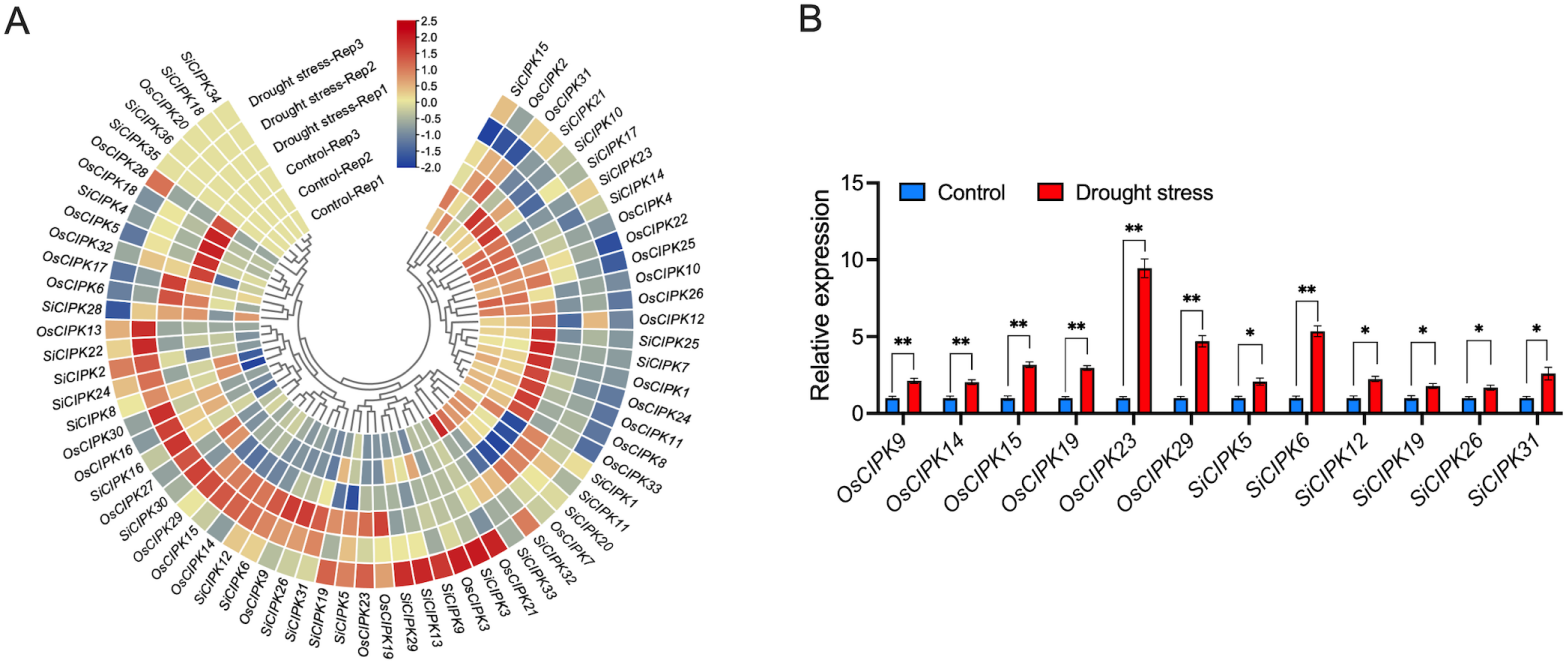
Expression analysis of the *CIPK* genes under drought stress conditions. **(A)** The expression analysis of *CIPK* genes in response to drought stress by RNA-seq. The heatmap was generated based on row-wise Z-score normalized log_2_(FPKM + 1) values. The color scale represents relative expression levels from low (blue) to high (red). **(B)** The expression analysis of *CIPK* genes in response to drought stress by qRT-PCR. n=3 biologically independent samples. The error bars represent ± SD. *p < 0.05, and **p < 0.01 compared to the Control (*t*-test).

Given the abundance of stress-responsive *cis*-elements identified in the promoter regions of *CIPK* genes, we investigated the expression patterns of these genes under abiotic stress conditions using RNA-seq and RT-qPCR. Comparative analysis of the *OsCIPK* and *SiCIPK* gene families in rice and foxtail millet revealed both conserved and species-specific responses to salt (**Figure 9**), cold (**Figure 10**) and drought (**Figure 11**) stresses. Under salt stress conditions (**Figure 9**), both species showed significant upregulation of specific *CIPK* genes, though with distinct expression patterns. In rice, a large number of *CIPK* genes (including *OsCIPK9*, *OsCIPK10*, *OsCIPK15*, *OsCIPK19*, *OsCIPK21*, *OsCIPK23*, *OsCIPK29*, *OsCIPK31*, *OsCIPK32* and *OsCIPK33*) were markedly induced by salt treatment. In contrast, their orthologous genes in foxtail millet maintained stable expression levels under the same conditions, indicating potential functional divergence in their stress response mechanisms. Notably, both species exhibited similarly strong salt-responsive induction of *CIPK1*, *CIPK2*, *CIPK6*, *CIPK7*, *CIPK16*, and *CIPK24* genes, suggesting conserved roles for these family members in salt stress adaptation across the two cereal species.

Cold stress treatment revealed striking species-specific expression patterns (**Figure 10**; **Supplementary Figure S5A**). In rice, the expression of a large number of *CIPK* genes was regulated by low temperature. For example, 15 genes (including *OsCIPK2*, *OsCIPK7*, *OsCIPK8*, *OsCIPK9*, *OsCIPK11*, *OsCIPK15*, *OsCIPK16*, *OsCIPK17*, *OsCIPK18*, *OsCIPK19*, *OsCIPK24*, *OsCIPK26*, *OsCIPK27*, *OsCIPK31*, *OsCIPK32* and *OsCIPK33*) were dramatically upregulated in response to cold stress, whereas 7 genes (including *OsCIPK1*, *OsCIPK4*, *OsCIPK5*, *OsCIPK10*, *OsCIPK12*, *OsCIPK14* and *OsCIPK29*) were significantly downregulated. In contrast, the responses in foxtail millet were generally more moderate, with only a limited number of *CIPK* genes exhibiting significant expression changes in response to cold. Among these, *SiCIPK19*, *SiCIPK24*, *SiCIPK29*, *SiCIPK34* and *SiCIPK35* showed consistent upregulation, while *SiCIPK10*, *SiCIPK12*, *SiCIPK14*, *SiCIPK15* and *SiCIPK21* were downregulated. This differential regulation may underlie the distinct cold tolerance capacities observed between these two species. Foxtail millet, cultivated predominantly in colder northern regions, likely possesses enhanced basal cold tolerance, whereas rice, largely grown in warmer southern areas, may rely on inducible stress responses.

Under drought stress, rice *CIPK* genes exhibited significantly greater expression variation compared to their foxtail millet homologs (**Figure 11**; **Supplementary Figure S5B**). Specifically, drought stress substantially suppressed the expression of a large set of *CIPK* genes (*OsCIPK1*, *OsCIPK8*, *OsCIPK10*, *OsCIPK11*, *OsCIPK12*, *OsCIPK22*, *OsCIPK24*, *OsCIPK25*, *OsCIPK26* and *OsCIPK33*) but strongly induced *OsCIPK9*, *OsCIPK14*, *OsCIPK15*, *OsCIPK19*, *OsCIPK23* and *OsCIPK29* in rice. In contrast, most foxtail millet *CIPK* genes showed minimal expression changes under drought stress, with only *SiCIPK7* and *SiCIPK25* being noticeably downregulated. Notably, *SiCIPK5*, *SiCIPK6*, *SiCIPK12*, *SiCIPK19*, *SiCIPK26* and *SiCIPK31* were significantly upregulated, suggesting their potential specialized roles in foxtail millet drought adaptation. Overall, these expression patterns highlight important regulatory divergences between rice and foxtail millet *CIPK* genes, likely reflecting their adaptations to distinct ecological niches.

## 4 Discussion

Plants have evolved sophisticated calcium-based signaling networks that enable them to perceive and respond to various environmental stresses, such as nutrient deficiencies, drought, salinity and extreme temperatures (Edel et al., 2017; Lee and Seo, 2021; Tong et al., 2021). CIPK can form essential signaling modules with CBLs that translate calcium signatures into specific physiological responses (Tang et al., 2020). Some studies have demonstrated the roles of the *CIPK* family in mediating rice responses to abiotic stresses, including salt tolerance (Qin et al., 2024), drought resistance (Lu et al., 2022) and cold adaptation (Zhang et al., 2019). The identification and characterization of *CIPK* in rice have been previously reported, with earlier studies identifying 30 putative *OsCIPK* genes in the rice genome (Xiang et al., 2007). In the present study, we conducted a comprehensive and systematic bioinformatic analysis of the *OsCIPK* gene family using current high-quality rice genome data. Our findings not only provide extensive new details regarding *OsCIPK* genes but also highlight their functional diversity in abiotic stress responses. Crucially, through comprehensive comparative genomics of the *CIPK* gene family in rice and foxtail millet, we revealed both deep conservation and significant divergence in the organizational structure of the *CIPK* family across the two species. These findings offer new insights into how calcium signaling networks evolve to support grain environmental adaptation, providing potential targets for enhancing crop stress resistance through breeding or engineering strategies.

In foxtail millet, *SiCIPK* genes were systematically named according to the established nomenclature of their orthologs in rice (**Supplementary Figure S1**). Furthermore, a comparative analysis of the structural and evolutionary differences of the *CIPK* gene family in rice and foxtail millet was conducted, providing new insights into the evolutionary and functional diversification of *CIPK* genes in monocotyledonous plants. In addition, an analysis of *cis*-acting elements in the promoters revealed that rice and foxtail millet *CIPK* genes respond to abiotic stress differently (**Figure 8**). This highlights the functional diversity of the *CIPK* gene family in crop stress resistance. These findings contribute to our understanding of the *CIPK* gene family in monocotyledonous plants and provide new candidate genes and a theoretical basis for crop stress resistance breeding.

A comprehensive identification of *CIPK* genes in foxtail millet revealed 35 members, but it is noteworthy that *SiCIPK27*, the orthologous gene of *OsCIPK27* found in rice, is absent (**Figures 1**, **2**). This absence may stem from several evolutionary and functional scenarios. Firstly, the retention or deletion of genes is a relatively common occurrence during the process of species formation. Alternatively, the foxtail millet genome may harbor a highly divergent sequence that evaded detection by our stringent BLASTp and HMM criteria, though manual inspection of syntenic regions did not reveal any candidate genes with significant homology. The functional implications of this absence are intriguing. In rice, OsCIPK27 is among the smallest CIPK proteins (43.8 kDa) and shows reproductive tissue-specific expression, suggesting a specialized role in development. Its loss in foxtail millet may reflect relaxed selection pressure due to differences in reproductive strategies or environmental adaptation. Notably, foxtail millet CIPK exhibit complete conservation of the NAF domain (unlike rice, where OsCIPK4 lacks this domain), implying stronger constraints on calcium signaling fidelity. Beyond this, the differences of CIPK proteins are manifested primarily in protein length and sequence variation outside these core domains, which may influence partner specificity or regulatory properties. The absence of SiCIPK27 might be compensated by functional redundancy or neofunctionalization of other SiCIPK, which could have subfunctionalized to cover its roles.

The observed differences in gene expression patterns, protein localization, and regulatory elements highlight how calcium signaling networks have diversified to support the specific requirements of C_3_ and C_4_ photosynthesis. The greater prevalence of leaf-expressed *CIPK* in foxtail millet (10 genes) compared to rice (7 genes) likely reflects the more complex photosynthetic coordination required in C_4_ plants (**Figure 7**). Under abiotic stress conditions, rice *CIPK* genes exhibited strong responses to salt and cold stress, whereas foxtail millet *CIPK* were more responsive to drought, reflecting their ecological adaptations. It is noteworthy that the qRT-PCR validation was conducted within a uniform genetic background, whereas the integrated RNA-seq data were derived from multiple varieties. The absolute expression levels and magnitude of stress-induced *CIPK* gene induction may vary across genotypes. This variability highlights the impact of genetic background on the precise regulation of stress-response gene expression. Despite these genotypic differences, however, the overall expression trends of the differentially expressed genes identified by RNA-seq were broadly consistent with the qRT-PCR results, which provides qualitative support for their stress responsiveness.

The chloroplast localization predicted for specific CIPK isoforms in both species suggests conserved roles in photosynthesis-related calcium signaling, while the distinct sets of chloroplast-targeted isoforms indicate pathway-specific specialization of these signaling components (**Figure 6**). It is important to note that the subcellular localization patterns reported here are based entirely on computational predictions. While these in silico analyses provide valuable insights and are consistent with known functional roles, they require validation through further experimental study. Differences in promoter architecture also provide potential evidence in support of this evolutionary divergence, with foxtail millet *CIPK* showing greater enrichment in light-responsive elements while rice *CIPK* are more associated with drought/ABA-related motifs (**Figure 8**; **Supplementary Figure S4**). This regulatory divergence mirrors the distinct environmental challenges faced by these species - foxtail millet’s adaptation to high-light conditions versus rice’s need to manage water stress in flooded paddies. The divergent evolution of *CIPK* genes between rice and foxtail millet offers remarkable potential for biotechnological innovation to improve crop resilience.

Our comparative analysis reveals two particularly promising research directions with immediate translational value. First, the drought-responsive *cis*-element rich promoters from rice *CIPK* (**Supplementary Figure S4**) could be utilized to enhance expression of native stress-responsive *CIPK* in foxtail millet through targeted promoter swapping. Given foxtail millet’s inherent drought tolerance, such genetic engineering approaches could potentially lead to lines with enhanced stress tolerance, representing a promising strategy for advancing drought resilience in cereal crops. Conversely, the introduction of key foxtail millet *CIPK* genes into rice presents an exciting opportunity to transfer stress tolerance traits across photosynthetic types. These C_4_-derived genes may confer novel stress response capabilities to rice, particularly if their unique protein features (including complete NAF domain conservation) enable more robust calcium signaling under adverse conditions.

While the transfer of key foxtail millet *CIPK* genes into rice presents an exciting opportunity to enhance stress tolerance, several biological challenges must be considered. Such as, while promoter-swapping strategies are conceptually promising, ensuring the reconstituted stress-responsive expression pattern operates with the desired specificity and magnitude in a heterologous system requires careful validation. Off-target effects or constitutive over-expression driven by strong stress-inducible promoters could lead to unnecessary energy expenditure and growth penalties. Therefore, future work should not only focus on the functional characterization of these candidate genes but also employ strategies such as tissue-specific or stress-inducible promoters. Meantime, techniques like CRISPR/Cas-mediated editing of native promoters can be applied to minimize negative pleiotropic effects and ensure seamless incorporation into the plant’s existing regulatory networks.

## 5 Conclusion

Our study provides a comprehensive comparative genomics genomic and transcriptional characterization of the *CIPK* gene families in rice and foxtail millet, demonstrating their crucial roles in stress adaptation. We identified 33 *OsCIPK* and 35 *SiCIPK* genes, all containing conserved kinase domains but exhibiting distinct physicochemical properties, gene structures, promoter element and expression patterns. These findings reveal how calcium signaling components have diversified through evolution to support environmental adaptation in C_3_ and C_4_ cereals, providing valuable targets for genetic improvement of stress-resilient crop varieties.

## Data Availability

The original contributions presented in the study are included in the article/**Supplementary Material**. Further inquiries can be directed to the corresponding authors.
