# Supplementary material for: Comparative genomics and expression analysis of the CIPK gene family in rice (Oryza sativa) and foxtail millet (Setaria italica)
Source: Front Plant Sci. 2026 Jan 5;16:1710663. doi: 10.3389/fpls.2025.1710663 (PMC12812626; doi:10.3389/fpls.2025.1710663)
Supplement: Supplementary file 1 [file DataSheet1.pdf]

Supplementary Materials

Title: Comparative Genomics and Expression Analysis of the *CIPK* Gene Family in Rice (*Oryza sativa*) and Foxtail Millet (*Setaria italica*)

Zhao Hu[1, 2, 4*], Run Qian[3, 4], Fengpu Xie[3], Ziwei Wang[1], Pingmei Yan[1*], Jing Yang[2*]

[1] College of Biological Sciences and Technology, Taiyuan Normal University, Taiyuan, China
[2] College of Life Science, Nanchang University, Nanchang, China
[3] School of Traditional Chinese Materia Medica, Shenyang Pharmaceutical University, Shenyang, China
[4] These authors contributed equally to this work.

* Correspondence:
Jing Yang
yangjing@ncu.edu.cn
Pingmei Yan
yanpingmei1968@163.com
Zhao Hu
zhaohu@tynu.edu.cn

Supplementary Figures

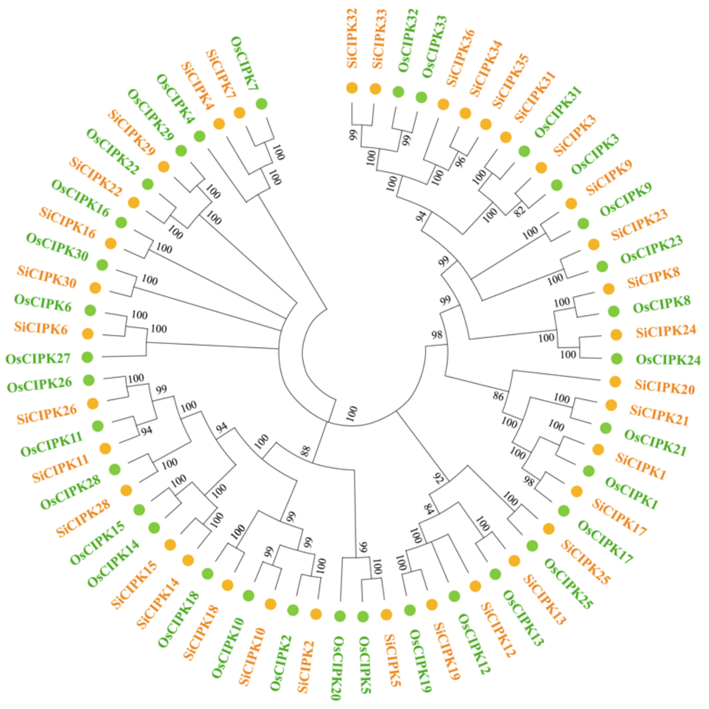

Figure S1 Phylogenetic relationships analysis of *CIPK* in rice and foxtail millet.

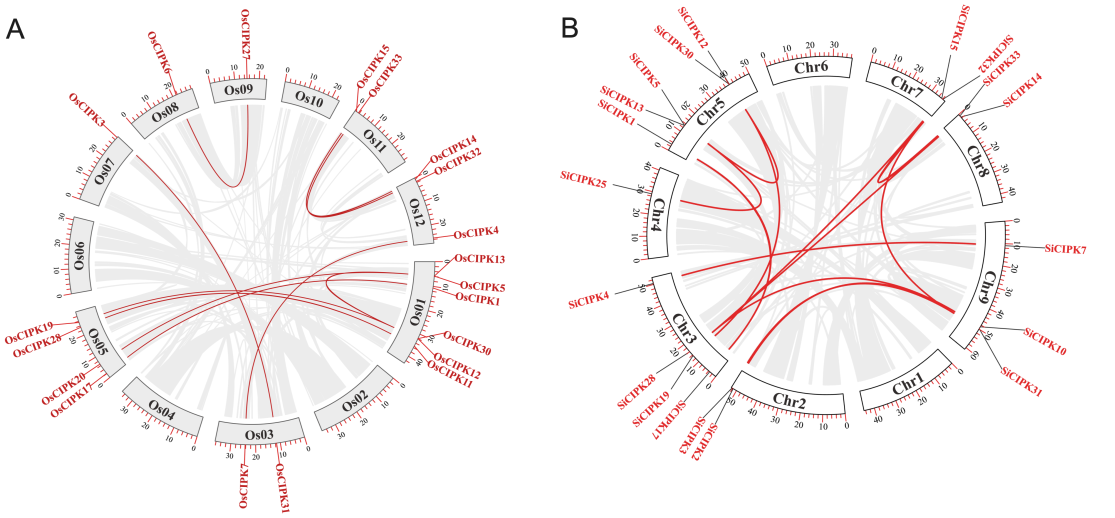

Figure S2 Gene segmental duplication analysis of *CIPK* in rice (**A**) and foxtail millet (**B**). Gray lines indicate all synteny blocks in the rice/ foxtail millet genome and the red lines indicate duplicated *CIPK* gene pairs.

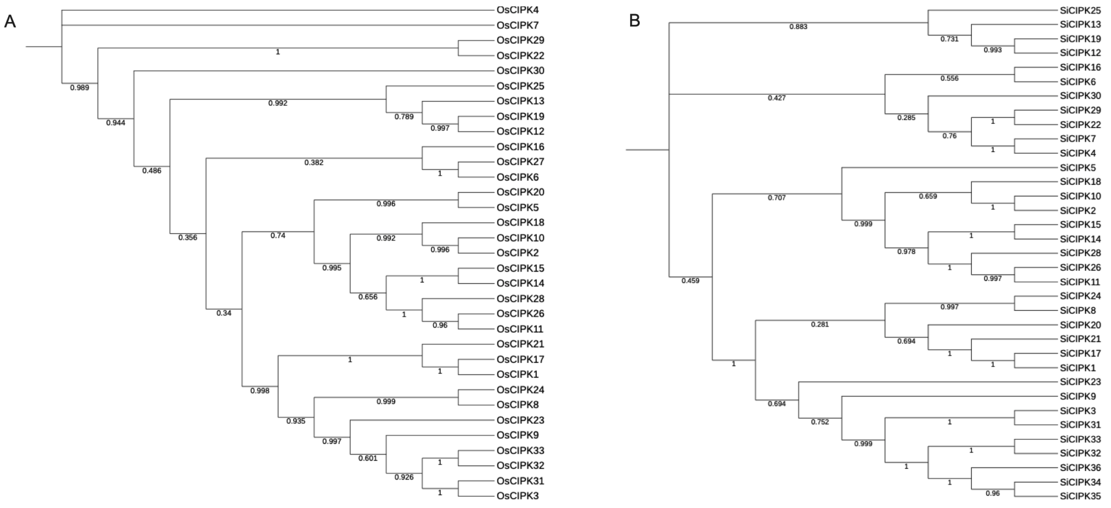

Figure S3 Phylogenetic relationships analysis of *CIPK* in rice (**A**) and foxtail millet (**B**) by maximum-likelihood (ML) method.

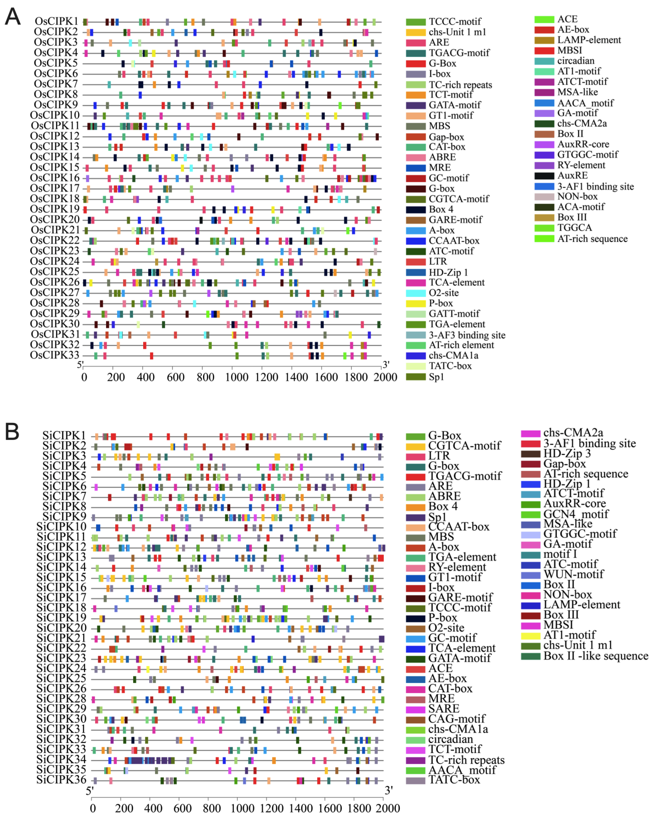

Figure S4 The distribution of these *cis*-regulatory elements across the *OsCIPK* (**A**) and *SiCIPK* (**B**) promoters. Promoter sequences (-2Kb) of *CIPK* were analyzed by PlantCARE. Different *cis*-elements are represented by different colors.

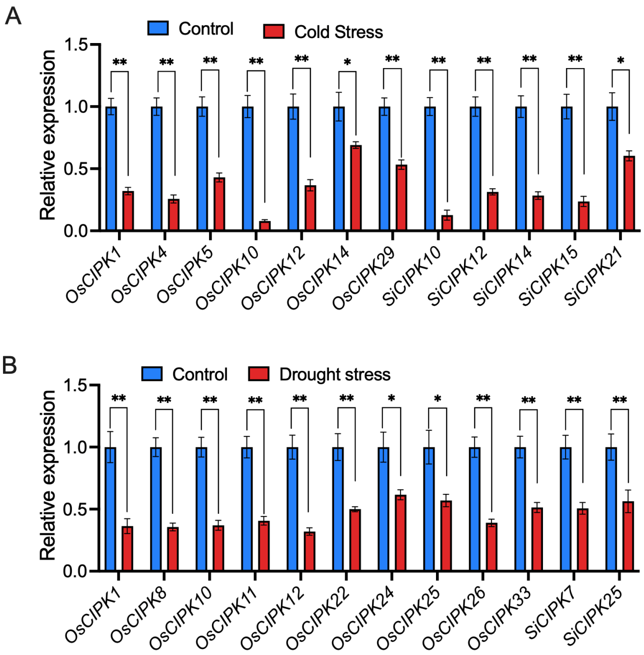

Figure S5 The expression analysis of *CIPK* genes in response to cold (**A**) and drought (**B**) stress by qRT-PCR. n=3 biologically independent samples. The error bars represent ± SD. * $p < 0.05$, and ** $p < 0.01$ compared to the Control (*t*-test).

Supplementary Tables

Table S1 qPCR primers used in this study

Gene	Forward primer (5'-3')	Reverse primer (5'-3')	Primer efficiency
OsActin1	ACCATTGGTGCTG AGCGTTT	CGCAGCTTCCATT CCTATGAA	103.2%
OsCIPK1	CTAGAGAGCTTCG ACAGCGG	CCAACAGACGGGC AAGAAGA	100.8%
OsCIPK2	ACATAGTCTGGGC TTGGCAG	CAAGAGGAACAA AAGCTGCAGAA	101.5%
OsCIPK4	GAGCAGCTGAGAA TGGAGCTA	TCCCTTGCCATTT ACAAACACC	102.4%
OsCIPK5	AGAGCATCCATCA CTGGCAC	GCCCTAAGCCTGA TGCTCAA	103.7%
OsCIPK6	TCGAGTACCGCTC ATTCTGC	TGCACGTCGCTAT CGAATCA	104.3%
OsCIPK7	TGCGATGGAGGGA AGAAAGAC	CTTTGACACAACA ATGCCACA	105.6%
OsCIPK8	AGCGCTAGTCTGC TTTGACA	ACCTGCAATAGTG GTGAGCA	106.4%
OsCIPK9	AGCGTTTGCACCA TTTCTCC	CGCTCCCTTTTGC AAGGATT	107.8%
OsCIPK10	TTGCAATTTCGGTT GGCTGG	GGGTACAAACCGG AGAAGCA	108.5%
OsCIPK11	CGTTGTTGCCACA AGTACCA	TCAGGAGTAACAC TCGGAGG	109.7%
OsCIPK12	CCGGCGATTTCAT ATGGCTC	AATTGGCACGGTG GGGAATA	100.2%
OsCIPK14	GCAGCAGTCGCAA GAGTAGA	AGCTTGAAAGGCT CAAAGCCA	101.1%
OsCIPK15	ATAGCAGCAGTCG CAAGAGT	GCTTGAAAGGCTC AAAGCCAAT	109.2%
OsCIPK16	AGTTCAACAAGTT CTGCGCC	TACAGATTTCACG TCGCGCT	100.5%
OsCIPK17	TCAGAACAGCAAT GCAGGGA	ACCACTCGGTTCT GTACACTG	101.8%
OsCIPK18	ATGCAGAGATCTT CGAGGTGAC	TTCACCTTGCCAA GCCCAAA	102.9%
OsCIPK19	TTATGTATGCCGC AGCAGTG	ACGATGTTTCCAC AACACCAG	103.8%
OsCIPK21	AGAGCCCCCAATA CAACGAC	TAGCCAATTTGCC CAGGAGG	104.9%
OsCIPK23	CATCGGAGGTCAC CGTGATT	GCCACAGGCCATT CCTTAGTA	105.7%

OsCIPK24	ACGTCGAAATGCT CTCGTGT	TTGTGGTAGGCAG GTTTCCC	106.5%
OsCIPK25	TTCTGCGACAACG AGCTCAA	ACAGCAACAGTGC CACATGA	107.3%
OsCIPK26	GGGGTTCTGAGGT TGGTAGC	CTGTGGCTGCTGT GATGGTA	102.3%
OsCIPK27	TGGACGTCAAGAA GGATGGC	GTTGCTACGGACA AACGAGC	108.1%
OsCIPK29	GTGAGTGATGTCT CCCGTCG	CTCTCGTCCACAA GAGAGGC	109.5%
OsCIPK30	AAGCGCTCTTGAT ATGCCGA	AATCTGACAGCGG AATGCCA	100.9%
OsCIPK31	AAGAAGGCAAAGG GGGACAC	CTACTCTACGGCG AACACCC	101.7%
OsCIPK32	GGCGACACCCTCG AATTTCA	AGTTTACAGGGCC ACTCACA	102.6%
OsCIPK33	CAAAAGGCGACAC CCTCGAA	CCATTCCTCCTCTC CCAAGT	103.9%
*Si*Actin	CAGTGGACGCACA ACAGGTAT	AGCAAGGTCAAG ACGGAGAAT	104.5%
SiCIPK1	ATCACGGGTATCA AACAGCAT	TTGGGAGAACTAA ACCAGGAAG	105.3%
SiCIPK2	TTACAAACAAAGG ATGCCCC	GATTCTTCAAACA TACCCGACA	106.2%
SiCIPK5	GTTGGCACAGTCA CAGGGTT	CCTTTGGAGTGCG AGATGAT	107.1%
SiCIPK6	ATACTTCAGGCAG CTCGTCT	CGAAGTCGACAAC CTTGAGG	108.7%
SiCIPK7	GGAGATGTTGGAG GTGTCGG	TCTTCGCAGACGT GCCAC	103.5%
SiCIPK10	GAGATTTCGGTGA TGAAGTTGG	CTCCTCCCTTGAC GTATTCTAAC	104.7%
SiCIPK12	TTGACACCAATCC GAACACTC	ACAGGCCATCCTC ATCATCTAT	105.9%
SiCIPK14	GAGGTTGGTGGCA CATAAGAA	TGCACTAATAAGC TGCTGGAAG	106.8%
SiCIPK15	GGGTTTGATCTATC TGGCCTCT	ATTCCTTCCCTCCT TCCTCC	107.6%
SiCIPK16	GCTACGCCAAGAT GTACCAGAA	CGAAGCCTTTCCT GAACCAC	108.4%
SiCIPK19	TCCAGCCACATTG TCATCAG	AAACCTCACTTCG TTGCCTC	109.8%
SiCIPK21	GATGTCCTCCTGCC TTGATTTA	GCTTGCCATTGCT CTTATGTACT	100.3%

SiCIPK24	TGGTCCTCTTATGA TGAATGCC	ATCCTGTTTGGTG ATGTGCCT	105.2%
SiCIPK25	GAGCAAATGCGGT TCGTGT	CGATGCTCACCTG GTACTCCT	106.3%
SiCIPK26	TTGGAGTATGCTA AAGGTGGTG	CATCTAGGAGTAG GTTTTCTGGC	107.2%
SiCIPK29	CTTGCGATGTTGA GACGAGC	TGCCCGTTGACAT CCATTT	108.3%
SiCIPK31	GATGGAGGCTATG ATGGTGC	CCAAGAGGGGCA GGTAAACT	109.1%
SiCIPK34	CATATGCGTGTAC GACCCCA	GCAGGACGTAAGT GCAGAGA	100.6%
SiCIPK35	GGCAAGGAGGTAC TTCCAACA	GCCCAGCCCAAAC TCAGATA	101.4%

Table S2 The information of RNA-seq datasets

BioProject ID	Species	Cultivar	Dev_stage	Tissue	Treatment
PRJNA827493	*Oryza sativa*	Nipponbare	seedling	leaf	cold stress
PRJNA1037192	*Oryza sativa*	not reported	seeding	leaf	Salt stress
PRJNA306542	*Oryza sativa*	Nipponbare	seeding	leaf	Drought stress
PRJNA1155684	*Setaria italica*	Chigu 26	five leaf stage	leaf	Drought stress
PRJNA805389	*Setaria italica*	Hong gu 2000	seeding	leaf	Salt stress
PRJNA767196	*Setaria italica*	not reported	3 weeks	leaf	cold stress

Table S3 Gene information of *OsCIPK* and *SiCIPK*

Rice		Foxtail millet	
Gene symbol	Gene ID	Gene symbol	Gene ID
OsCIPK1	*Os01g0292200*	*SiCIPK1*	*Seita.5G031200*
OsCIPK2	*Os07g0678600*	*SiCIPK2*	*Seita.2G432000*
OsCIPK3	*Os07g0687000*	*SiCIPK3*	*Seita.2G439100*
OsCIPK4	*Os12g0603700*	*SiCIPK4*	*Seita.3G380200*
OsCIPK5	*Os01g0206700*	*SiCIPK5*	*Seita.5G145900*
OsCIPK6	*Os08g0441100*	*SiCIPK6*	*Seita.6G163200*
OsCIPK7	*Os03g0634400*	*SiCIPK7*	*Seita.9G162500*
OsCIPK8	*Os01g0536000*	*SiCIPK8*	*Seita.5G190200*
OsCIPK9	*Os03g0126800*	*SiCIPK9*	*Seita.9G557900*
OsCIPK10	*Os03g0339900*	*SiCIPK10*	*Seita.9G411700*
OsCIPK11	*Os01g0824600*	*SiCIPK11*	*Seita.5G364100*
OsCIPK12	*Os01g0759400*	*SiCIPK12*	*Seita.5G325500*
OsCIPK13	*Os01g0206300*	*SiCIPK13*	*Seita.5G145700*
OsCIPK14	*Os12g0113500*	*SiCIPK14*	*Seita.8G014400*
OsCIPK15	*Os11g0113700*	*SiCIPK15*	*Seita.7G300300*
OsCIPK16	*Os09g0418000*	*SiCIPK16*	*Seita.2G206200*
OsCIPK17	*Os05g0136200*	*SiCIPK17*	*Seita.3G053800*
OsCIPK18	*Os05g0332300*	*SiCIPK18*	*Seita.3G285000*
OsCIPK19	*Os05g0514200*	*SiCIPK19*	*Seita.3G181100*
OsCIPK20	*Os05g0208100*	*SiCIPK20*	*Seita.1G065400*
OsCIPK21	*Os07g0637000*	*SiCIPK21*	*Seita.2G405500*
OsCIPK22	*Os05g0334750*	*SiCIPK22*	*Seita.3G284500*
OsCIPK23	*Os07g0150700*	*SiCIPK23*	*Seita.2G032200*
OsCIPK24	*Os06g0606000*	*SiCIPK24*	*Seita.4G221700*
OsCIPK25	*Os06g0543400*	*SiCIPK25*	*Seita.4G175800*
OsCIPK26	*Os02g0161000*	*SiCIPK26*	*Seita.1G079400*
OsCIPK27	*Os09g0418500*	*SiCIPK28*	*Seita.3G205700*
OsCIPK28	*Os05g0476350*	*SiCIPK29*	*Seita.2G431900*
OsCIPK29	*Os07g0678300*	*SiCIPK30*	*Seita.5G325400*
OsCIPK30	*Os01g0759200*	*SiCIPK31*	*Seita.9G422900*
OsCIPK31	*Os03g0319400*	*SiCIPK32*	*Seita.7G311200*
OsCIPK32	*Os12g0132200*	*SiCIPK33*	*Seita.8G003400*
OsCIPK33	*Os11g0134300*	*SiCIPK34*	*Seita.8G045200*
		SiCIPK35	*Seita.8G171900*
		SiCIPK36	*Seita.8G172100*

Note: Due to the absence of a direct homologue of *OsCIPK27* within the foxtail millet, and to maintain consistency with rice nomenclature, the *SiCIPK27* position has been omitted.

Table S4 Ka/Ks analysis of *CIPK* gene in rice and foxtail millet

Seq_1	Seq_2	Ka	Ks	Ka/Ks
SiCIPK2	*SiCIPK10*	0.166255007	2.012318585	0.082618631
SiCIPK3	*SiCIPK31*	0.109930288	0.868089592	0.126634727
SiCIPK19	*SiCIPK12*	0.141976791	0.94444669	0.15032801
SiCIPK17	*SiCIPK1*	0.147851429	1.012223815	0.146065946
SiCIPK28	*SiCIPK15*	0.265887396	Undefined	Undefined
SiCIPK28	*SiCIPK14*	0.273083025	2.785815048	0.098026258
SiCIPK4	*SiCIPK7*	0.138738893	0.377183051	0.367829076
SiCIPK28	*SiCIPK10*	0.271490857	3.34462646	0.081172251
SiCIPK25	*SiCIPK13*	0.428134105	0.637071138	0.672035004
SiCIPK13	*SiCIPK12*	0.334676914	1.046495463	0.319807324
SiCIPK5	*SiCIPK30*	0.388013664	1.425869679	0.272124213
SiCIPK15	*SiCIPK14*	0.018581343	0.096995955	0.191568228
SiCIPK32	*SiCIPK33*	0.00882291	0.034819133	0.253392589
SiCIPK32	*SiCIPK31*	0.170247052	Undefined	Undefined
OsCIPK13	*OsCIPK12*	0.362228528	1.140476862	0.317611466
OsCIPK5	*OsCIPK30*	0.345134219	1.815920022	0.190060253
OsCIPK5	*OsCIPK20*	0.222425564	1.301267629	0.170929914
OsCIPK11	*OsCIPK28*	0.161615091	0.880474936	0.183554448
OsCIPK12	*OsCIPK19*	0.177320828	0.848244409	0.2090445
OsCIPK1	*OsCIPK17*	0.13546062	0.704043138	0.192403863
OsCIPK31	*OsCIPK3*	0.11365033	1.115820853	0.101853563
OsCIPK7	*OsCIPK4*	0.455333203	1.175243417	0.387437357
OsCIPK6	*OsCIPK27*	0.158041366	0.365753999	0.432097439
OsCIPK15	*OsCIPK14*	0.002480981	0.047607048	0.052113742
OsCIPK33	*OsCIPK32*	0.002948165	0.045508271	0.064783066

Table S5 Protein information of CIPK family

Gene name	AA	MW	PI	Instability Index	Aliphatic Index	GRAVY
OsCIPK1	461	52201.57	6.26	38.1	92.36	-0.403
OsCIPK2	443	50272.36	9.18	28.7	84.74	-0.421
OsCIPK3	445	50952.4	6.9	39.63	86.79	-0.392
OsCIPK4	439	47208.61	12.06	81.28	76.54	-0.631
OsCIPK5	461	51965.79	9.28	33.26	84.82	-0.402
OsCIPK6	451	48910.12	8.95	34.71	90.64	-0.18
OsCIPK7	447	48373.95	9.35	52.94	93.27	-0.148
OsCIPK8	406	46457.14	6.16	46.47	93.94	-0.346
OsCIPK9	389	43990.5	6.11	40.2	79.46	-0.357
OsCIPK10	439	50122.1	9.13	33.05	91.91	-0.341
OsCIPK11	502	56558.9	8.48	43.73	86.81	-0.448
OsCIPK12	540	59807.67	8.3	48.23	84.26	-0.285
OsCIPK13	511	56010.92	8.05	40.81	81.17	-0.214
OsCIPK14	439	50324.27	9.44	35.41	89.66	-0.408
OsCIPK15	434	49697.65	9.53	36.91	90.02	-0.398
OsCIPK16	456	50504.1	8.84	44.25	88.77	-0.169
OsCIPK17	454	50915.03	6.93	34.14	87.82	-0.395
OsCIPK18	457	51497.4	8.84	34.62	87.64	-0.348
OsCIPK19	508	56913.03	7.26	48.88	79.65	-0.392
OsCIPK20	466	51350.69	8.29	41.61	81.39	-0.303
OsCIPK21	444	50099.5	7.96	33.61	90.9	-0.376
OsCIPK22	451	49323.41	8.03	46.93	89.8	-0.214
OsCIPK23	449	50580.06	9.23	38.56	85.35	-0.401
OsCIPK24	408	46338.44	7.63	38.82	96.96	-0.194
OsCIPK25	514	56980.4	8.78	44.3	81.98	-0.301
OsCIPK26	493	55907.91	9.13	38.36	81.52	-0.526
OsCIPK27	404	43786.44	8.4	31.57	87.45	-0.116
OsCIPK28	435	49330.96	9.32	39.38	89.93	-0.353
OsCIPK29	443	48212.55	8.69	37.03	87.72	-0.127
OsCIPK30	476	53563.48	9.34	51.7	83.17	-0.362
OsCIPK31	449	50954.47	7.98	31.16	86.01	-0.414
OsCIPK32	438	50337.81	7.64	38.18	84.98	-0.492
OsCIPK33	438	50289.68	6.82	38.52	85.21	-0.495
SiCIPK1	463	52018.73	6.58	38.9	93.02	-0.319
SiCIPK2	450	51089.29	9.28	30.9	83.84	-0.444
SiCIPK3	447	50585.24	7.53	29.65	87.27	-0.34

SiCIPK4	436	47527.89	9.23	53.32	91.56	-0.182
SiCIPK5	450	51121.77	9.23	37.95	82.56	-0.429
SiCIPK6	451	48830.94	9.14	34.99	86.08	-0.197
SiCIPK7	431	46884.15	9.03	48.71	95.78	-0.078
SiCIPK8	450	50625.82	6.35	42.69	88.84	-0.291
SiCIPK9	452	50826.26	7.99	35.42	78.12	-0.408
SiCIPK10	449	51000.31	9.22	28.96	88.37	-0.316
SiCIPK11	508	57559.18	8.73	47.18	87.91	-0.473
SiCIPK12	518	57249.11	8.92	46.91	83.78	-0.26
SiCIPK13	521	57450.15	6.73	46.1	80.71	-0.319
SiCIPK14	445	50920.82	9.11	36.27	85.87	-0.457
SiCIPK15	444	50755.62	9.1	34.47	84.08	-0.463
SiCIPK16	473	51366.8	8.81	46.53	87.27	-0.125
SiCIPK17	472	52397.86	6.55	36.1	89.41	-0.322
SiCIPK18	459	51866.95	9.03	32.19	86.62	-0.348
SiCIPK19	402	45512.65	5.52	43.38	77.34	-0.416
SiCIPK20	442	49515.37	5.82	45.37	91.52	-0.268
SiCIPK21	444	49910.43	8.56	29.85	93.54	-0.33
SiCIPK22	439	48403.3	8.98	46.22	86.92	-0.257
SiCIPK23	449	50702.29	9.28	32.39	83.81	-0.416
SiCIPK24	452	50843.64	8.52	34.21	94.47	-0.183
SiCIPK25	479	52658.41	8.78	40.69	85.53	-0.206
SiCIPK26	463	53143.07	9.06	37.96	87.6	-0.51
SiCIPK28	416	47024.03	9.6	40.42	94.74	-0.207
SiCIPK29	432	46742.59	7.68	40.74	91.06	-0.061
SiCIPK30	476	53079.17	9.22	41.93	86.47	-0.311
SiCIPK31	449	50743.37	8.25	31.76	88.2	-0.367
SiCIPK32	440	50461.1	8.05	37.82	82.41	-0.477
SiCIPK33	440	50353.01	7.66	39.18	85.95	-0.452
SiCIPK34	433	49635.14	7.61	39.85	93.63	-0.332
SiCIPK35	981	111880.43	5.85	54.28	84.27	-0.474
SiCIPK36	435	50295.98	6.82	40.29	94.99	-0.345
